# Relaxation of mucosal fibronectin fibers in late gut inflammation following neutrophil infiltration in mice

**DOI:** 10.1038/s44341-024-00006-y

**Published:** 2025-02-04

**Authors:** Ronja Rappold, Konstantinos Kalogeropoulos, Gianna La Regina, Ulrich auf dem Keller, Emma Slack, Viola Vogel

**Affiliations:** 1 Institute of Translational Medicine, ETH Zurich, Zurich, Switzerland; 2 Institute of Food, Nutrition and Health, ETH Zurich, Zurich, Switzerland; 3 Department of Biotechnology and Biomedicine, Technical University of Denmark, Kgs. Lyngby, Denmark; 4 Botnar Research Center for Child Health, Basel, Switzerland

**Keywords:** Diseases, Molecular biophysics, Imaging, Mass spectrometry

## Abstract

The continuously remodeled extracellular matrix (ECM) plays a pivotal role in gastrointestinal health and disease, yet its precise functions remain elusive. In this study, we employed laser capture microdissection combined with low-input proteomics to investigate ECM remodeling during *Salmonella*-driven inflammation. To complement this, we probed how fibronectin fiber tension is altered using a mechanosensitive peptide probe. While fibronectin fibers in healthy intestinal tissue are typically stretched, many lose their tension in intestinal smooth muscles only hours after infection, despite the absence of bacteria in that area. In contrast, within the mucosa, where *Salmonella* is present starting 12 h post infection, fibronectin fiber relaxation occurred exclusively during late-stage infection at 72 h and was localized to already existing clusters of infiltrated neutrophils. Using N-terminomics, we identified three new cleavage sites in fibronectin in the inflamed cecum. The unique, tissue layer-specific changes in the molecular compositions and ECM fiber tension revealed herein might trigger new therapeutic strategies to fight acute intestinal inflammation.

## Introduction

In recent years, the prevalence of inflammatory gastrointestinal diseases has exhibited a concerning upward trend^1^. An understudied aspect of the underlying mechanisms of such diseases is the extracellular matrix (ECM) within the intestinal environment, which offers considerable potential for novel interventions. The ECM is a complex network of proteins and proteoglycans that plays a pivotal role in tissue integrity, maintenance, remodeling, and repair^2–4^. In the context of inflammatory gastrointestinal diseases, the ECM is integral to both tissue destruction and repair processes^5,6^.

The intestine is a remarkably complex organ, encompassing multiple tissue layers. These layers play vital roles in maintaining intestinal barrier integrity, facilitating nutrient absorption, regulating the immune system, and orchestrating digestion^7^. The intestinal mucosa serves as the primary interface for interactions with luminal content and forms a barrier against pathogens^8^. Meanwhile, the muscularis externa, a smooth muscle layer surrounding the intestinal tube, is responsible for gut motility^9^. This intricate system is stabilized by a fibrillar ECM network that is composed of a large number of tissue-specific ECM elements which contain binding sites for cell receptors, enzymes and signaling factors that together regulate tissue homeostasis, growth, and repair^10–12^. Not only the biochemical composition but also physical factors of the ECM are affecting its reciprocal crosstalk with the embedded cells, and thus its overall functions, including its viscoelastic properties and tensile strength^13–15^. Major ECM remodeling processes are happening in the matrix during inflammation^6,16^ and as recently reported can even precede clinical symptoms of intestinal inflammation^17^, further emphasizing the need for molecular examination of the ECM’s role in gastrointestinal health.

Fibronectin, an essential ECM protein, facilitates cell adhesion, migration, and differentiation as well as tissue growth and wound healing^11,18,19^. Fibronectin is essential for viability, as knock-out mice do not survive beyond embryonic day 8, which highlights its critical role but also complicates functional studies^20^. Fibronectin is mechanosensitive, showing altered functions depending on the fiber’s tensile state^21,22^. Fibronectin fiber stretching thus can activate cryptic binding sites, inaccessible in the mechanically relaxed protein, which are required for fibronectin fibrillogenesis during fibronectin matrix assembly, or destroy multivalent binding sites^15^. Importantly, its interactions with integrins^23,24^, collagens^25^, interleukin-7^26^ and tissue transglutaminase 2^27^, were all reported to be dependent on the tensile state of fibronectin. Despite its functional implications investigated in vitro, the tensional state of fibronectin fibers is mostly unresolved in ex vivo or in vivo settings, primarily due to the lack of appropriate tools. We have previously developed^28,29^ and validated a fibronectin fiber tension probe FnBPA5^30–32^. This peptide probe is derived from the bacterial fibronectin adhesin of *Staphylococcus aureus* and presents a powerful tool to measure fibronectin fiber tension in ex vivo cryosections^32^. FnBPA5 binds with nanomolar affinity, via a multivalent network of backbone hydrogen bonds, to the N-terminal FnI_2-5_ domains of fibronectin exclusively in a low tensional state. In stretched fibronectin fibers the distance between these four FnI domains is increased, thereby destroying the multivalent binding motif such that the FnBPA5 peptide cannot bind any longer with high affinity^32^. Notably, the FnBPA5 probe cannot distinguish between fibers that are structurally relaxed due to enzymatic cleavage, or due to the presence of other load-bearing ECM elements, such as more rigid collagen fiber bundles^25^.

Here, we investigated the ECM mechanobiology in the oral streptomycin mouse model of *Salmonella enterica* subspecies *enterica* serovar Typhimurium (*S*. Tm) infection. This is a highly reproducible model with a well-resolved and reproducible timeline of inflammation^33,34^, which allows mapping of new findings to defined steps in the inflammatory response against the invading bacteria^35^. A plethora of host defense mechanisms including secretion of antimicrobial peptides, expulsion of infected epithelial cells, recruitment of neutrophils and other inflammatory cells are involved in infection control^34,36–38^. This limits the systemic dissemination of *S*. Tm. However, these defense mechanisms also drive extensive tissue damage (e.g. epithelial cell expulsion, tissue layer reorganization, edema formation) in the intestine as well as collateral damage to the commensal microbiota^39,40^.

In this study, we employed laser capture microdissection coupled to data independent acquisition (DIA) tandem mass spectrometry (LC-MS/MS) to investigate tissue layer-specific changes in ECM in inflammation. In addition to common infection control mechanisms, our analysis revealed protein expression and ECM remodeling differences during the host response across the individual tissue layers. Importantly, we present for the first-time evidence of a spatial correlation between the relaxation of fibronectin fibers, which succeeds the infiltration of neutrophils in the inflamed intestine. These findings shed new light on the intricate interplay between mechanical forces, ECM remodeling and immune response altering protein expression, as well as fibronectin fiber tensional state in a highly defined manner. Finally, we have measured tissue layer-specific proteomes for both the healthy and *Salmonella*-infected mouse cecum, identified three new cleavage sites in fibronectin in the inflamed cecum, and present these datasets as a resource to the field.

## Results

### Spatial proteomics analysis of healthy and severely inflamed mouse ceca

**Fig. 1:**
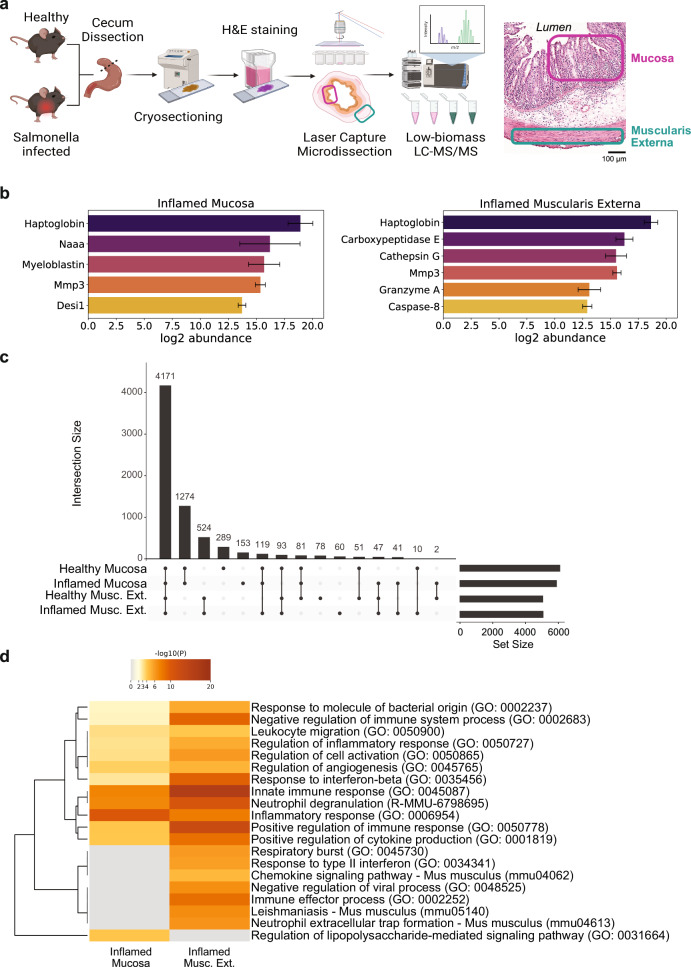
Cross-sectional spatial protein map of the mouse cecum in health and acute inflammation. **a** Schematic of workflow for tissue layer-specific proteomics analysis using laser capture microdissection followed by data-independent acquisition LC-MS/MS. Mucosal and muscularis externa tissue areas were extracted from H&E-stained cryosections from *S*. Tm or mock-infected mice. Mucosa and muscularis externa regions indicated in an inflamed H&E-stained cross-section on the right. Illustration generated using BioRender.com. **b** Relative abundances of proteases identified solely in inflamed mucosa (left) and muscularis externa (right) tissue. Error bars represent standard deviation of mean values (*n* = 3-5 mice). Desi1, desumoylating isopeptidase 1; Naaa, N-acylethanolamine-hydrolyzing acid amidase; MMP3, matrix metalloproteinase 3; **c** Upset plot representing the distribution of detected proteins in the four biological conditions tested (Healthy Mucosa, Inflamed Mucosa, Healthy Muscularis Externa, Inflamed Muscularis Externa. **d** Functional Gene Enrichment Analysis of proteins detected solely in the inflamed mucosa and the inflamed muscularis externa reveal strong inflammatory response in both layers with different key areas. H&E, hematoxylin and eosin; LC-MS/MS, liquid chromatography tandem mass spectrometry; Musc. Ext., Muscularis Externa; *S*. Tm, *Salmonella enterica* subspecies *enterica* serovar Typhimurium.

To elucidate the tissue layer-specific proteome of healthy and severely inflamed mouse cecum, we employed a laser capture microdissection (LCM) approach coupled to high sensitivity LC-MS/MS-based proteomics. The LCM technique facilitated targeted isolation of discrete regions within the cecal tissue, offering an unprecedented level of spatial resolution. Infection with 5×10^7^ colony forming units (CFU) of *S*. Tm by voluntary feeding, resulted in high intestinal (10^9^ CFU / g of cecum content) as well as systemic counts ( ~ 10^5^ CFU / g of spleen) of *S*. Tm bacteria from day 1 post infection (Supplementary Fig. 1)^33,41^. Mucosa and muscularis externa tissue areas were selectively collected for both healthy and inflamed (day 3 post infection (p.i.)) conditions using LCM as depicted in Fig. 1a. To confirm the specificity of the LCM tissue isolation, we compared the relative abundances of proteins specific for the mucosa and muscularis externa. Chromogranin A (Chga), a marker for gastrointestinal endocrine cells, was exclusively detected in the mucosa. Both Mucin2 and Mucin13, primarily expressed in epithelial cells exhibited higher abundance in the mucosa. However, since these proteins are also present in endothelial cells, which are partially found in the muscularis externa, the difference in their abundance between the two tissues was less pronounced compared to other proteins. Conversely, calponin 1 (Cnn1), desmin (Des), and transgelin (Tagln) showed a significantly higher abundance in the muscularis externa (all p < 0.05), as they are known to be specifically expressed in smooth muscle cells (Supplementary Fig. 2). In total we detected 7668 proteins, 57% of which were ubiquitously found in all four biological conditions (healthy mucosa, healthy muscularis externa, inflamed mucosa and inflamed muscularis externa). Further, we identified more proteins specific for the mucosa compared to the muscularis externa, independent of the health status (Fig. 1c).

### Functional analysis of proteins detected solely in the inflamed conditions

In total 56 proteins were detected only in the inflamed mucosa (Supplementary Data 1), while 124 proteins were found only in the inflamed muscularis externa (based on at least three biological replicates) (Supplementary Data 2). Functional analysis using Metascape^42^ confirmed the involvement of these proteins in the immune response against bacterial infection (Fig. 1d). Interestingly, many highly significant gene ontology (GO) terms involved in immune response presented an increased significance in the muscularis externa or were even found solely in this tissue layer (GO: 0002252 - immune effector process and GO: 0035456 - response to interferon-beta, respectively). Identification of lipopolysaccharide signaling (GO: 0031664 - regulation of lipopolysaccharide-mediated signaling pathway) specifically in the mucosa reflects the location of invasive bacteria at this timepoint of the infection^37,43^. Remarkably, we detected laminin subunit gamma 2 (Lamc2) only in the inflamed mucosa, which might suggest that the epithelial basement membrane gets partially degraded, whereas it remains below the detection limit in the healthy mucosa. Laminins are important structural components of the epithelial basement membrane^44^. Lamc2 has been associated with epithelial cell migration following cleavage by matrix metalloproteinase 2 in wound scenarios^45^ and was reported to be upregulated in dextran sulfate sodium induced intestinal inflammation^46^. Investigating the implications of upregulated Lamc2 in the inflamed intestinal mucosa could provide valuable insights into tissue responses and might even be used for therapeutic applications in the future. We also detected interesting protease candidates likely involved in ECM remodeling: in mucosa and muscularis externa, we could detect matrix metalloproteinase 3 (MMP3), a matrix remodeling metalloproteinase known to create a pro-inflammatory 70 kDa fibronectin fragment^47^, and haptoglobin, which has antimicrobial and antioxidant activity and is involved in hemoglobin clearance and the immune response^48,49^. Specifically for the inflamed muscularis externa, we identified cathepsin G, known for its ECM remodeling functions and potential fibronectin cleavage capability^50^, and granzyme A, a serine protease with recently demonstrated ECM remodeling function^51^ (Fig. 1b).

### Protein abundances change in a tissue layer-specific manner upon *S*. Tm-induced inflammation

**Fig. 2:**
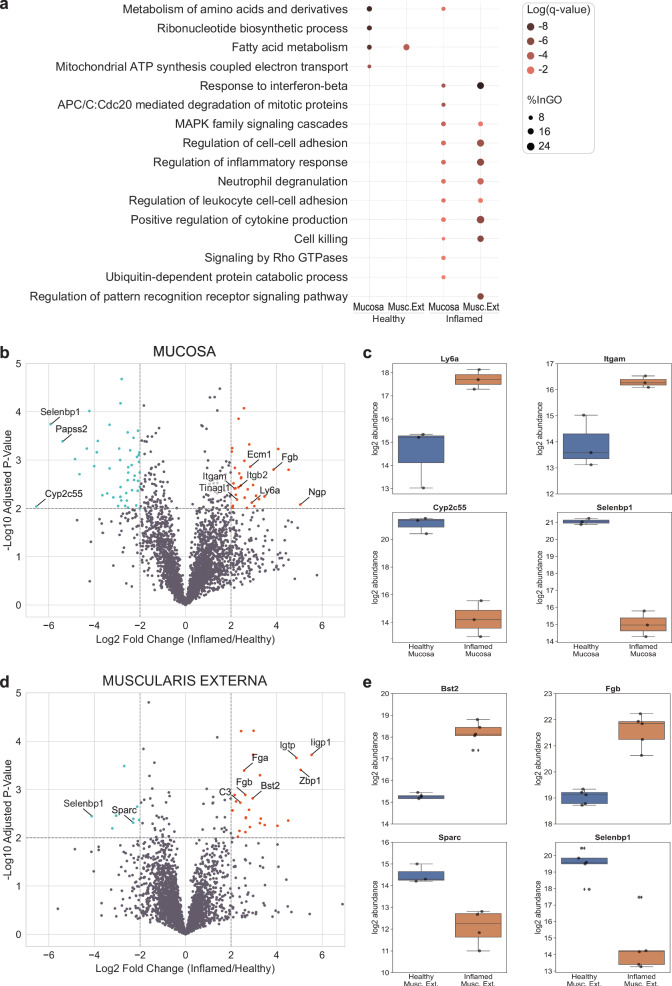
Differential expression of proteins upon inflammation in mucosa and muscularis externa in metabolism, inflammatory response and proteolytic activity. **a** Dotplot of functional gene enrichment analysis representing interesting GO terms for up- and downregulated proteins in the two tissue layers. The color of the dots is representing the significance and their size indicates the percentage of proteins from the group mapping with the functional category. **b** Volcano plot of proteins differentially expressed in the mucosa. **c:** Individual abundance plots of interesting mucosal proteins that were differentially expressed upon inflammation. **d** Volcano plot of proteins differentially expressed in the muscularis externa. **e** Individual abundance plots of interesting proteins in the muscularis externa that were differentially expressed upon inflammation. The Sparc signal was only detected in 3 (healthy) and 4 (inflamed) mice out of the 5 as indicated by the marker points. **b** + **d** Level for significant fold change: log_2_FC ≥ 2. Significance level: -log_10_(adj.p-val) ≥ 2. Upregulated proteins are marked in orange, downregulated proteins in lightblue. **c** + **e** Boxplots extend from first quartile to third quartile with marker points representing individual mice (*n* = 3–5) and median abundance indicated as horizontal line. The whiskers represent the largest/lowest datapoint of the dataset that falls within 1.5× the interquartile range. Musc. Ext., Muscularis Externa.

In addition to the proteins exclusively detected in the inflamed conditions, we found many proteins exhibiting differential expression between the inflamed and healthy states. Predominantly upregulated proteins within the mucosa were associated with the inflammatory response (e.g. Sting1 and Stat1), including functions such as cytokine production, neutrophil degranulation, and mitogen-activated protein kinase (MAPK) family signaling (Fig. 2a). Notably, a substantial downregulation of metabolism-related proteins (e.g. Ass1 and Auh) in the mucosa (R-MMU-71291 - Metabolism of amino acids and derivatives) was evident, underscoring a metabolic shift associated with inflammation. Building on these observations, our focused analysis within the mucosa revealed 33 proteins experiencing significant upregulation (log_2_FC ≥ 2 and -log_10_ p-value ≥ 2), while 48 proteins underwent significant downregulation in the inflamed state compared to the healthy tissue layer (Fig. 2b, Supplementary Data 3). In the mucosa, neutrophilic granule protein and the lymphocyte antigen 6 family member A (Ly6a) are specifically upregulated (Fig. 2b, c), reflecting the strong infiltration of neutrophils into this tissue area. Additionally, expression of the leukocyte-specific integrin subunits β2 and αM is increased (Fig. 2d), likely reflecting the invasion of immune cells^52,53^. Several of the detected proteins are also upregulated in other inflammatory intestinal diseases: extracellular matrix protein 1 (ECM1), which was reported to be elevated in inflammatory bowel disease^54^ and tubulointerstitial nephritis antigen like 1 (Tinagl1), a matricellular protein with protease activity upregulated in gastric cancer and involved in MMP regulation^55^. As a representative instance of downregulated metabolism in the inflamed mucosa (Fig. 2e), the decreased expression of cytochrome Cyp2c55 is noteworthy, as this underscores the broader metabolic shift within the inflamed setting.

In the muscularis externa, we observed no significant upregulation of leukocyte-specific integrins. Instead, functional analysis unveiled the downregulation of fatty acid metabolism upon inflammation (R-MMU-8978868 – Fatty acid metabolism) and the upregulation of similar inflammatory response pathways as observed in the mucosa. Specifically, we detected 26 significantly upregulated (log_2_FC ≥ 2 and -log_10_ p-value ≥ 2) and 8 downregulated proteins in the inflamed condition compared to the healthy one (Fig. 2d, Supplementary Data 4). One interesting protein upregulated in this tissue layer was Bst2, also known as tetherin or CD317 (Fig. 2e), which is a lipid raft associated protein with potential ECM-cell interaction function^56^ and which is expressed in response to interferons. Muscularis externa-specific downregulation was observed for the matricellular glycoprotein Secreted protein acidic and rich in cysteine (Sparc) (Fig. 2e). Sparc is known for its ability to promote collagen fibril assembly, by stabilizing microtubule networks via integrin-linked kinase binding to integrins^57,58^, and its upregulated expression was shown in aging mice to induce the inflammatory interferon-response in macrophages, promoting their conversion from anti- to pro-inflammatory^59^. Reports on Sparc regulation in intestinal disorders are still limited with a recent study showing increased colonic mucosal mRNA levels in active ulcerative colitis^60^. This interesting discrepancy between chronic and acute intestinal inflammation and tissue layers (as well as human vs. mouse) is intriguing but its functional understanding goes beyond the scope of this study. Additionally, we detected proteins that were differentially regulated in both tissue layers (mucosa and muscularis externa). Fibrinogen beta chain (Fgb) was detected to be upregulated (Fig. 2b–c, e) showcasing the importance of the blood clotting system. Selenium-binding protein 1 (Selenbp1, also known as methanethiol oxidase) was downregulated upon severe inflammation (Fig. 2b–e). Selenbp1 is found in mature enterocytes catalyzing the conversion of methanethiol to hydrogen sulfide and other components. It was also reported to be downregulated in various cancers associated with poor prognosis^61,62^.

### The distribution of identified matrisome proteins is consistent across both tissue layers and disease states

We noticed variations in the expression of integrins and ECM remodeling proteins, including MMP3, cathepsin G, and members of the serpin family. This sparked our interest in understanding the distinctions within the matrisome related proteins, across the four biological groups. Intriguingly, our analysis revealed that the percentage of detected matrisome components remained consistent across all four conditions (3–5%) (Supplementary Fig. 3a). Furthermore, the distribution of the individual subcategories (proteoglycans, glycoproteins, collagens, ECM remodeling factors, ECM-affiliated proteins, and secreted factors) exhibited no significant changes between the tissue layers and the physiological state (Supplementary Fig. 3b). Given that ECM remodeling can be more subtle than changes in these overarching categories, we additionally checked abundance levels of the individual ECM components fibronectin, collagen I, tenascin C and MMP3 in the four different conditions (Supplementary Fig. 3c). Fibronectin, collagen I and tenascin C are present at higher levels in the muscularis externa compared to the mucosa in general but remain similar during inflammation. MMP3, which was only detected in the inflamed tissue layers showed similar abundance levels in the two tissue layers. Considering our identification of elevated MMP3 levels - a well-known protease capable of cleaving the ECM component fibronectin - we directed our focus towards exploring potential alterations in fibronectin fibers within the two distinct tissue layers.

### Probing healthy cecal tissue using a fibronectin fiber tension probe

**Fig. 3:**
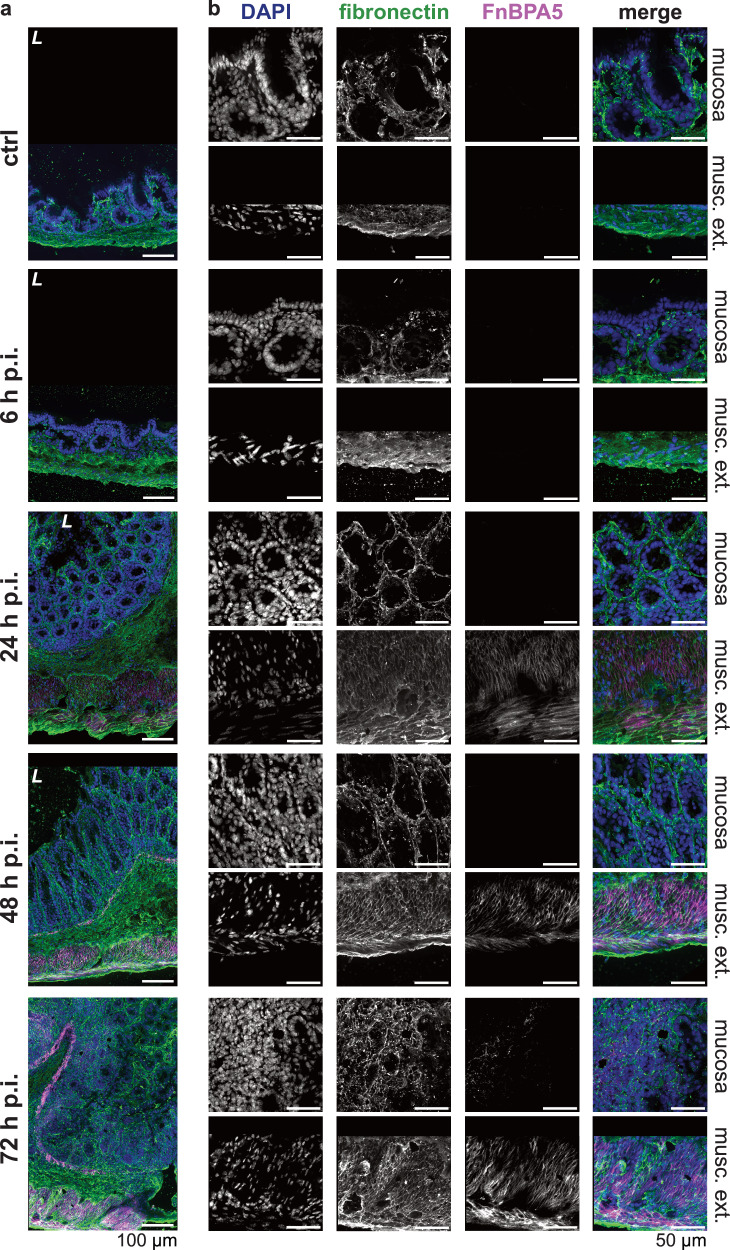
Relaxation of fibronectin fibers in inflamed cecum in the two tissue layers mucosa and muscularis externa over the timecourse of the *S*. Tm infection. **a** Representative images of immunohistochemistry stainings of total fibronectin using a polyclonal anti-fibronectin antibody (green), relaxed fibronectin fibers using Cy5.5-FnBPA5 (magenta) as well as cell nuclei using DAPI (blue) at all four timepoints (6 h p.i., 24 h p.i., 48 h p.i., and 72 h p.i.) and in the mock-infected mice (ctrl), demonstrate FnBPA5 signal in the muscularis externa at 24 h p.i. and in the mucosa at 72 h p.i. (*n* = 5 mice per group). Scale bar: 100 µm. **b** Higher resolution zoom-ins of the same tissues for mucosa and muscularis externa highlights the kinetics of fibronectin fiber relaxation in the two cecal tissue layers. Scale bar: 50 µm. p.i., post infection; musc. ext., muscularis externa; L, lumen.

Fibronectin, known for its broad functions regulating tissue growth, remodeling and repair processes^11,18,19^ and the mechano-sensitivity of its binding sites, partially activated or destroyed by fiber stretching^15^, is an interesting candidate to compare ECM remodeling during *S*. Tm infection-induced intestinal inflammation. Additionally, previous research revealed highly tensed fibronectin fibers in other healthy organs, while progressive loss of tension in tumor stroma^30,31^. This motivated us to probe the conformational state of fibronectin fibers in the healthy as well as the inflamed intestine. To study the tensional state of fibronectin fibers, we applied the bacterially derived FnBPA5 peptide, which identifies relaxed fibronectin fibers via binding of the N-terminal fibronectin domains FnI_2-5_ in a mechanosensitive manner^28,29^. To control for non-specific peptide binding we applied a scrambled peptide version with a shuffled amino acid sequence^32^. Fibronectin and FnBPA5 co-staining showed abundant fibronectin signal across all tissue layers, but no presence of low tensional state fibronectin fibers in healthy ceca (Fig. 3a, b, top row). This observation is in line with previous observations in other healthy organs showing low to no FnBPA5 binding^30^. We recently reported similar results in the context of bacterially induced intestinal edema (strong expansion of the submucosal layer), where fibronectin fiber degradation and thus relaxation seemed to be inhibited by an excess of protease inhibitors^63^.

### Fibronectin fiber tension in the smooth muscle layers strongly decreases early during *S*. Tm infection

**Fig. 4:**
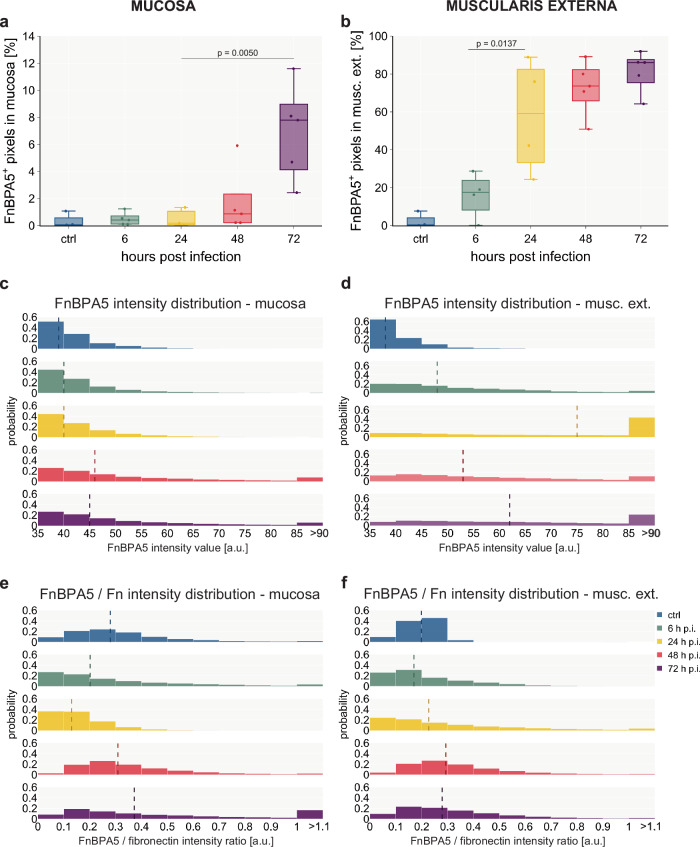
Quantification of mucosal and muscularis FnBPA5 signal during the timecourse of the *S*. Tm infection shows an increase in both tissue layers with the muscularis externa experiencing earlier and more severe fibronectin fiber relaxation. **a** Quantification of FnBPA5^+^ pixels in the mucosa yields a median rate of 8% of FnBPA5^+^ pixels at the late stage of the infection, while earlier days only show very low levels **b:** Quantification of FnBPA5^+^ pixels in the muscularis externa demonstrates a strong increase from the beginning of the inflammation, resulting in more than 80% of FnBPA5^+^ pixels at 72 h p.i. **a** + **b** FnBPA5^+^ pixels defined as pixels with intensity above the negative control value. Boxplots extend from first quartile to third quartile with median indicated as horizontal line and marker points representing individual mice (*n* = 3–5). Statistical analysis: One-way ANOVA with Tukey’s multiple comparison test, p-values indicated. **c** + **e** Histograms of intensity distributions of FnBPA5^+^ pixels **(c)** (ctrl, 6 h, 24 h, 48 h, 72 h p.i. conditions from top to bottom) and FnBPA5/fibronectin intensity ratios **(e)** demonstrate that there is not only an increase in number of FnBPA5^+^ pixels but also a signal intensity increase of those in the late stage of the infection in the mucosa. **d** + **f** Histograms of intensity distributions of FnBPA5^+^ pixels **(d)** and FnBPA5/fibronectin intensity ratios **(f)** verify that there is not only an increase in number of FnBPA5^+^ pixels but also an increase in signal intensity. **c–f** Dashed vertical lines represent median intensity values. Pixel-by-pixel data pooled from at least 3 images from 3-5 mice per timepoint each. Fn, fibronectin.

At early timepoints, we observed that the FnBPA5 tension probe only bound in the smooth muscle cell-containing muscularis layers: the muscularis mucosa (located between the mucosa and the submucosa) and the muscularis externa. FnBPA5 staining becomes prominently visible in mice 24 h p.i. as illustrated in Fig. 3. However, even at the 6 h p.i. timepoint, a discernible increase in signal intensity can be quantified through pixel-by-pixel analysis (Fig. 4b). During the timecourse of the *Salmonella* infection, the muscularis signal increases dramatically and results in more than 80% of FnBPA5-positive pixels on day 3. Visualizing the distribution of the FnBPA5 pixel intensities clearly shows few high-intensity pixels on day 1, shifting to broadly increased fluorescence intensity on days 2 and 3 (Fig. 4d). The increasing FnBPA5 intensity in the muscularis externa can also be observed in the intensity distributions of the ratio of FnBPA5 normalized to fibronectin pixel values (Fig. 4f). This may represent fibronectin relaxation due to the overall muscle contraction induced by the inflammatory response. The cecum shrinkage and its morphological changes during *S*. Tm infection have been described histopathologically already in 2003^33^ and recently more quantitatively and with a focus on ECM remodeling by us^63^. Of note, we controlled for post-mortem muscle changes as 1) no FnBPA5 signal is observed in the healthy intestinal muscularis and 2) ceca were manually filled with mounting medium prior to embedding to avoid non-physiological tissue collapse.

### Mucosal fibronectin fiber tension decreases in spatially defined areas and only in the late stage during the *S*. Tm infection

In the mucosa, the FnBPA5 signal and thus fibronectin fiber tension changes are only detectable at late timepoints. First on day 2 p.i. sporadic distinct small pixel clusters of FnBPA5 appear. On day 3 the FnBPA5 signal becomes prominent forming larger clusters in the mucosa (Fig. 3b, bottom row). The average FnBPA5-positive pixel coverage in the mucosa stays low in comparison to the muscularis externa signal. On day 2 p.i., we observed a slight increase and on day 3 the FnBPA5-positive mucosal tissue area accounts for 4–9% of the total mucosal area (Fig. 4a). As can be seen in the intensity histograms, the FnBPA5 pixel intensity shows higher values on day 2 and day 3 compared to the control and the early timepoints of the infection representing the altered number of available binding sites for the FnBPA5 peptide on relaxed fibronectin fibers (Fig. 4c). The ratio of FnBPA5 and fibronectin pixel intensity remains low until day 2, where it shows a slight increase. By day 3, high ratios are observed, representing a higher fraction of relaxed fibronectin fibers (Fig. 4e).

At least two mechanisms could explain why fibronectin fibers lose their tension: fiber cleavage or mechanical reinforcement of the tissue through the deposition of collagen bundles. In tumor sections, FnBPA5 was reported to be in spatial proximity with collagen fiber bundles as visualized by second harmonic generation (SHG)^30^. In contrast to cancer stroma, the measured SHG signal in the inflamed mucosa at day 3 turned out to be quite low, and we did not observe any elevated SHG signal in the locally elevated mucosal FnBPA5 regions (Supplementary Fig. 4). This indicates that the fibronectin fiber relaxation in the inflamed mucosa is not caused by thick collagen fibers taking over as force bearing elements^25^. To further validate this, we performed immunohistochemistry stainings against collagen I and applied the collagen hybridizing peptide (CHP), indicative of denatured collagen helices. The collagen I staining was homogeneously distributed throughout the mucosa and no spatial correlation of increased collagen and the FnBPA5-positive mucosal tissue areas could be detected (Supplementary Fig. 5a, b). However, the CHP signal on day 3 p.i. was increased in the FnBPA5-positive mucosal areas hinting towards elevated proteolytic processes ongoing in these spatially confined areas (Supplementary Fig. 5c).

### Fibronectin fiber relaxation is spatially but not temporally correlated with neutrophil cluster occurrence

**Fig. 5:**
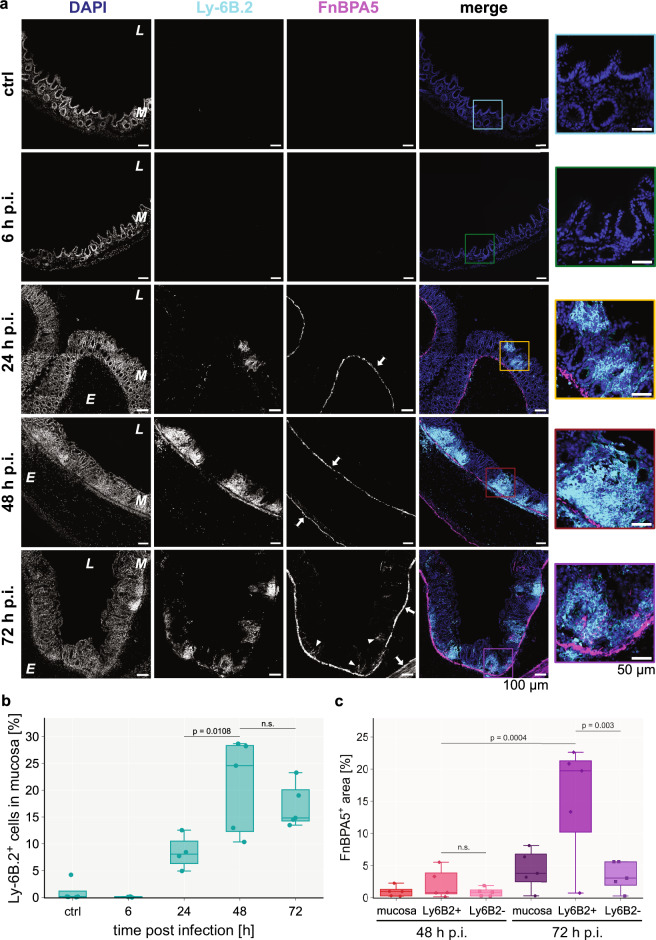
Neutrophil clusters are adjacent to regions rich in relaxed fibronectin fibers (FnBPA5) in the mucosa on day 3 but not on day 2. **a** Representative images of immunohistochemistry stainings of neutrophils using an anti-Ly6B.2 antibody (cyan), relaxed fibronectin fibers using Cy5.5-FnBPA5 (magenta) as well as cell nuclei using DAPI (blue) during the timecourse of the *S*. Tm infection (top to bottom: control mouse, 6 h, 24 h, 48 h and 72 h p.i.), show an increase of infiltrating neutrophil clusters in the mucosa from 24 h p.i. onwards but importantly the mucosal FnBPA5 signal is only increasing at 72 h p.i. (arrowheads). In contrast, the FnBPA5 signal in the muscularis mucosa and muscularis externa (arrows) is visible from 24 h p.i. (confirming results shown in Fig. 3). Color-coded zoom-in overlay images for regions of interest are shown for every timepoint. Scale bar: 100 µm. Scale bar zoom-in: 50 µm. **b** Quantification of percentage of Ly6B.2^+^ cells in the mucosa during the timecourse demonstrates increasing numbers of neutrophils peaking at 48 h p.i. (25%) but keeping high (15%) levels of tissue neutrophils at 72 h p.i. **c** Quantification of FnBPA5^+^ pixels in spatial relation to neutrophil clusters reveals strong spatial correlation between them at 72 h p.i. At 48 h p.i., the percentage of FnBPA5^+^ pixels is comparable between mucosal areas with and without neutrophil clusters, while at 72 h p.i. the percentage of FnBPA5^+^ pixels in the neutrophil cluster regions is increased approximately 4-fold compared to areas without neutrophil clusters. **b–c** Marker points represent individual mice (*n* = 3–5 mice per group with at least 10 images taken per mouse). Boxplots extend from first quartile to third quartile with median indicated as horizontal line. The whiskers represent the largest/lowest datapoint of the dataset that falls within 1.5× the interquartile range. Statistical analysis: One-way ANOVA with Tukey’s multiple comparison test, p-values indicated, n.s.: *p* ≥ 0.05. p.i., post infection.

Neutrophil invasion into the mucosa^64^ as well as their swarming behavior^65^ are well-known phenomena and our proteomics data corroborated this by exhibiting enhanced leukocyte infiltration via the presence of neutrophilic granule protein and Ly6a (Fig. 2b). Thus, we contemplated the possible involvement of neutrophils in the fibronectin fiber relaxation process in the inflamed mucosa. Neutrophils are not only known as the first line of defense against bacterial infections, but also secrete a lot of proteases upon activation, many of which are known to be able to cleave fibronectin^66^. Staining with an anti-Ly6B.2 antibody showed appearance of first clusters of neutrophils in the mucosa after 24 h p.i. (Fig. 5a, Supplementary Fig. 6b). On day 2 the number of neutrophils significantly increased (p = 0.0108) and stayed at high levels on day 3. The number of Ly6B.2-positive cells increased to 25% of the total mucosal cells on day 2 (Fig. 5b, Supplementary Fig. 6b). We then quantified the FnBPA5 signal specifically in Ly6B.2-positive cluster regions. Additional microscopy images for day 2 and 3 are shown in Supplementary Fig. 7. The Ly6B.2-positive regions have been automatically annotated by the image analysis software QuPath^67^ using a thresholding-based user-trained algorithm (Supplementary Fig. 6a). Strikingly, we observed a significant increase of FnBPA5-positive pixels (p = 0.003) as well as FnBPA5 mean intensity (p = 0.0084) in these regions compared to Ly6B.2-negative mucosal areas only on day 3, but not on day 2 (Fig. 5c, Supplementary Fig. 6c). The percentage of FnBPA5-positive tissue area accounted for 20% in the Ly6B.2-positive clusters, while it maintained low levels in the Ly6B.2-negative regions comparable to the overall mucosa (around 5%). Thus, the mucosal FnBPA5 signal is spatially strongly correlated with neutrophil clusters in the late stage of the infection. Since the area fraction of these regions is small in comparison to the whole mucosal area, the localized FnBPA5 signal does not significantly affect the averaged mucosal FnBPA5 tissue coverage. This indicates that maturing neutrophil clusters (day 3 p.i.), observed in the cecal mucosa of *S*. Tm infected mice, are closely associated with fibronectin fiber relaxation either directly or indirectly. Neutrophils commonly recruit and cluster with other cell types, especially monocytes and macrophages during inflammation^68^; thus, this effect may be directly mediated by neutrophils through the secretion of specific proteases, reactive oxygen species (ROS) release or extracellular trap formation, or indirectly driven by the collective response of multiple cell types to severe inflammation and collateral tissue damage in these regions.

### Spatial proteomics analysis of FnBPA5-positive regions reveals upregulation of aminopeptidase N and tenascin C

**Fig. 6:**
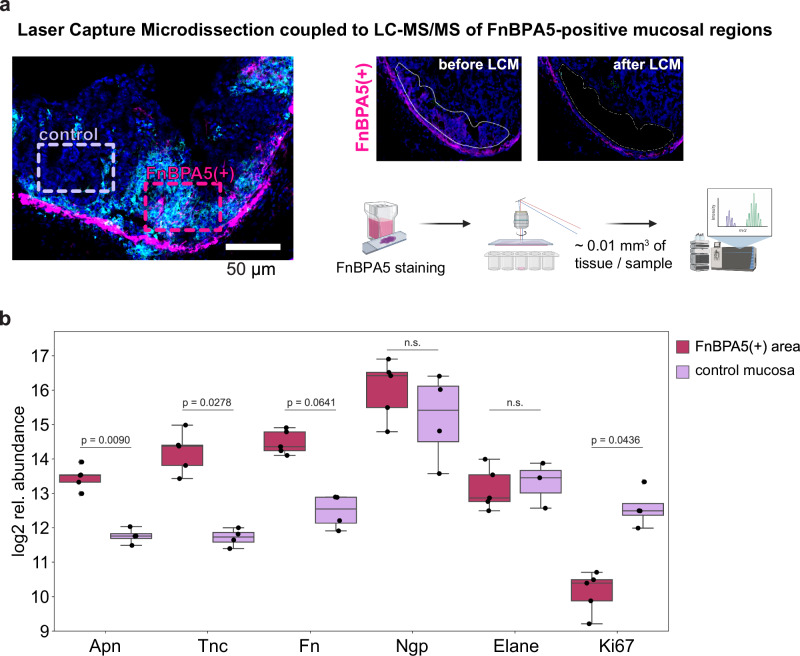
Identification of differentially expressed proteins in FnBPA5-positive mucosal regions. **a** Schematic of LCM collection of FnBPA5-positive and FnBPA5-negative regions from the mucosa. Illustration generated using BioRender.com **b** Abundances of interesting differentially expressed proteins demonstrate significantly higher levels of aminopeptidase N (Apn) and tenascin C (Tnc) in the FnBPA5-positive regions. Statistical analysis: One sided student t-test, p-values corrected for multiple testing with Benjamin-Hochberg FDR correction, p values indicated, n.s.: p ≥ 0.05. Fn, fibronectin; Ngp, neutrophilic granule protein; Elane, neutrophil elastase.

To further elucidate this phenomenon, we set out to investigate the specific spatial proteome of FnBPA5-positive cluster areas in inflamed ceca on day 3. We collected these tissue regions using LCM and conducted an additional low biomass LC-MS/MS experiment (Fig. 6a). Our analysis revealed significant upregulation (p = 0.0090) of aminopeptidase N (Apn) in the FnBAP5-positive areas compared to FnBPA5-negative regions in the mucosa (Fig. 6b). Apn, also known as CD13, is a widely expressed transmembrane metallopeptidase with numerous functions, including activation of matrix metalloproteinases, signal transduction and immune response^69,70^. Previous studies have reported that Apn can degrade extracellular matrix components^71,72^. Additionally, Apn interacts with NGR motifs in ECM proteins, indicating that it plays a role in cell motility and adhesion by directly interacting with the ECM and more specifically also fibronectin^73^. Moreover, we observed significant upregulation of tenascin C, a critical ECM component involved in cell adhesion, in the FnBAP5-positive regions. This finding is particularly notable as the overall mucosal tenascin C levels did not change during inflammation (Supplementary Fig. 3), underscoring the spatial specificity of tenascin C expression in the inflamed mucosa. Neutrophilic granule protein as well as neutrophil elastase abundances were not altered between FnBPA5-positive and -negative regions. Additionally, we observed a trend towards higher fibronectin levels and a significant reduction of Ki67 abundance, indicative for decreased epithelial cell proliferation, in the FnBPA5-positive areas (Fig. 6b).

### Terminal amine isotopic labeling of substrates identifies 3 unknown cleavage sites in fibronectin type III domains elevated in the inflamed cecum

**Fig. 7:**
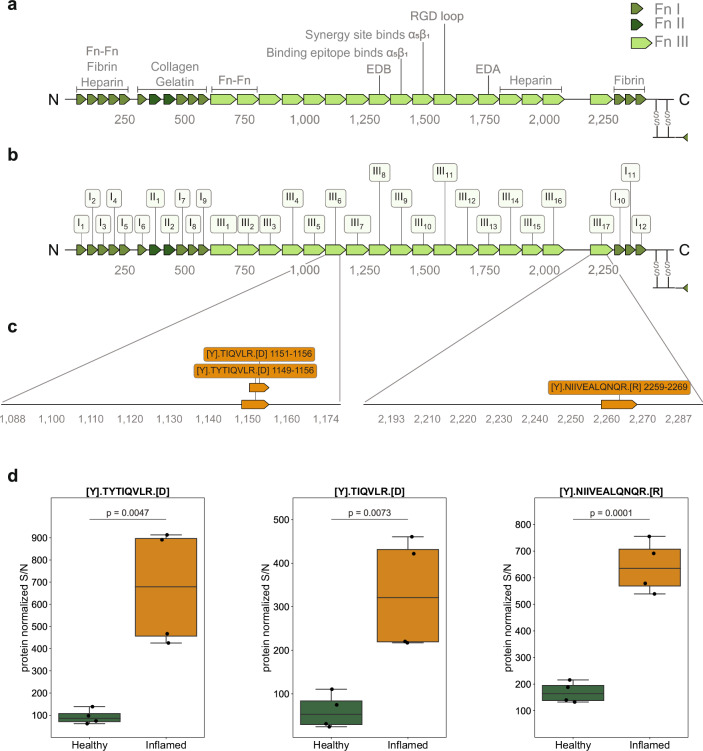
Identification of three new peptides and corresponding cleavage sites in fibronectin in the inflamed cecum using TAILS analysis. **a** Schematic illustration of fibronectin's sequence with individual FnI, FnII and FnIII domains and binding regions indicated. **b** Schematic illustration with domain order and labeling adopted from UniProt: FnIII_8_ corresponds to EDB, FnIII_13_ corresponds to EDA. **c:** Sequence of peptides significantly enriched in the *S*. Tm infected cecum and their annotations on the fibronectin protein. **d** Relative abundances of the three identified peptides elevated in the inflamed cecum. Boxplots extend from first quartile to third quartile with marker points representing individual mice (2 mice per condition in technical duplicates) and horizontal lines the median abundance. The whiskers represent the largest/lowest datapoint of the dataset that falls within 1.5× the interquartile range. Statistical analysis: One sided student t-test, p-values corrected for multiple testing with Benjamin-Hochberg FDR correction, p-values indicated Fn, fibronectin.

To analyze putative proteolytic cleavage events in healthy versus severely inflamed cecum tissue, we utilized the terminal amine isotopic labeling of substrates (TAILS) workflow. With this method it is possible to detect both natural protein N-termini and protease generated neo-N-termini by labeling protein N-termini prior to the depletion of internal tryptic peptides^74,75^. While degradomics analysis of LCM derived tissue areas would have provided spatially resolved insights into fibronectin cleavages, state of the art workflows still require microgram quantities of protein, which are impossible to obtain with LCM (which typically yields a few ng of protein). Therefore, we used whole cecum tissue (including all tissue layers) for the TAILS analysis and included two healthy mice and two severely inflamed mice (day 3 *S*. Tm infection) in technical replicates. Following LC-MS/MS analysis, the data processing was performed using CLIPPER 2.0, a novel tool integrating annotation, statistics, and visualization of degradomics datasets^76^. In total, we identified 3407 proteins and 2939 N-termini from the cecal samples. 827 of these N-termini were acetylated, while 2112 were non-acetylated (Supplementary Fig. 8). We detected 101 unique N-termini that were significantly elevated in the inflamed cecal tissue. Amongst those were interesting peptides from S100-A9, complement C3 and haptoglobin. The haptoglobin cleavage site at (R(102).(103)IIGGSM) is located right in between the haptoglobin alpha and beta chain, hinting at induced activation of its important functions ranging from antibacterial activity to hemoglobin capturing. In the healthy cecal tissue we detected 162 upregulated N-termini with peptides in cathepsin B and meprin A subunit beta, indicating a higher activation in the healthy state. In the context of this work, we further focused on fibronectin cleavage to investigate if there might be significant cleavage events in the inflamed conditions, which could possibly explain the increase in FnBPA5 signal. Indeed, we found 3 new peptides to be significantly higher represented in the inflamed cecum compared to the healthy one (Fig. 7d). Two of them are located in fibronectin type III domain 6 (sequence positions Thr^1149^ - Arg^1156^ and Thr^1151^ - Arg^1156^) and one is located in fibronectin type III domain 17 (sequence position: Asn^2259^ - Arg^2269^) (Fig. 7a–c). All 3 N-terminal cleavage sites (P1 position at Tyr^1148^, Tyr^1150^ and Tyr^2258^, respectively) have not been reported before and cannot be mapped to specific proteases. Interestingly though, all three cleavage sites have tyrosine in their P1 position, which indicates potential chymotryptic-like protease activity. Follow-up experiments will be necessary to understand which specific proteases are responsible for these fragments and whether these peptides have specific functions in the severely inflamed cecum.

## Discussion

The need for enhanced understanding and improved treatment options for gastrointestinal diseases is of central relevance. Investigating ECM alterations during intestinal inflammatory processes provides a novel and promising avenue for addressing this necessity. Here, we combine a well-studied intestinal inflammation model, mechanosensitive imaging, and LCM-coupled MS-based proteomics. With this approach, we identify spatiotemporally definite ECM and proteome changes occurring within the two tissue layers mucosa and muscularis externa during *S*. Tm induced intestinal inflammation. Of equally important significance, we discover and describe three new cleavage sites in fibronectin using degradomics. Though a protein specificity motif search did not allow for the identification of candidate proteases, this protease must be upregulated in the inflamed cecum. Two potential candidates, which we detected in our LCM dataset would be myeloblastin (also known as proteinase 3) and cathepsin G, both of which exhibit chymotryptic-like cleavage specificity. We detected myeloblastin exclusively in the inflamed mucosa and cathepsin G in the inflamed muscularis externa (Fig. 1b). Another interesting candidate would be chymase 1, which shares the same cleavage specificity and is expressed in mast cells which are elevated in intestinal *S*. Tm infected intestine^77,78^. The tyrosine residues, common to all three motifs, might also get attacked by ROS, leading to chemical modifications, which might then promote cleavage^79^.

While both tissue layers exhibit a common set of differentially expressed proteins indicative of a general inflammatory response against the *S*. Tm infection, distinct characteristics (e.g. regulation of lipopolysaccharide-mediated signaling pathway for the mucosa vs. response to type II interferon specific for muscularis externa) (Fig. 1d) and individual protein expression responses (e.g. upregulation of Lamc2 vs Bst2) (Supplementary Data 1, Fig. 2e), are evident. In the muscularis externa, we found cytokine production as well as cell-killing processes enhanced compared to the mucosa emphasizing its distinct role in the response against the *S*. Tm bacteria. Going beyond the control of bowel movement, the involvement of the muscularis externa is increasingly recognized by others (e.g. secretion of factors stimulating epithelial regeneration and proliferation^80^) and is in accordance with our finding showing a strong response to interferons. Interestingly, we found the upregulation of individual proteases to be very distinct between the tissue layers. A strong upregulation of proteases was observed with varying spatial distribution of MMPs in the inflamed intestinal tissue in a published *Salmonella* fibrosis model (e.g. MMP7 upregulated in epithelium, cathepsin D in the inflammatory infiltrate, others in both)^81^. This highlights that in acute (day 3) as well as in persistent *Salmonella* infection (day 21) the controlled spatial expression of proteases is important in the immune response and could potentially be used for the development of targeted therapeutics.

One surprising finding in our study was the early onset, followed by a much more pronounced relaxation of fibronectin fibers in the muscularis externa compared to the delayed effects seen in the mucosa. The low tensional state fibronectin fibers in the smooth muscle layers are most probably linked to the shrinkage of the organ itself, which has been extensively reported previously^33,40,82^. Cecal volume reduction must logically be associated with or even driven by the contraction of the smooth muscle layers. The performed pathway enrichment analysis of the N-terminomics dataset indeed identified smooth muscle contraction as a significant term enriched in inflamed cecum (Supplementary Fig. 5e). Furthermore, proteases involved in extracellular matrix remodeling such as cathepsin G and granzyme A, which exhibit capability to cleave fibronectin and other ECM fibers, were detected solely in the muscularis externa. Taken together, this discovery could hint towards the possibility of fibronectin fiber cleavage as a contributing factor to promoting the fibronectin fiber tension loss and thus the strong FnBPA5 signal in the muscularis layers.

The observed substantial downregulation of metabolism-related proteins, upregulation of leukocyte-specific integrins as well as neutrophil markers specifically in the inflamed mucosa, but not in the muscularis externa, confirms the highly inflamed state of the mucosa. Our study revealed an unexpected distinct spatial pattern of fibronectin fiber relaxation in areas near neutrophil clusters within the mucosa, at the late stage of *S*. Tm infection (day 3). This finding raises additional questions about highly localized protease activity and its functional consequences in the severely inflamed mucosal surrounding. The temporal dynamics observed, where neutrophil clusters are abundant on day 2, yet the FnBPA5 signal is only evident on day 3, suggest possible shifts in gene expression within these clusters leading to increased protease release targeting fibronectin. The detected upregulated aminopeptidase might be involved in fibronectin fiber relaxation; however further characterization is required to confirm this role. Proteolytic degradation could hold interesting downstream functions as it is known that cleaved ECM fibers and more specifically also fibronectin fragments have important and different functions than their full-length counterparts^83–85^. The additional observation of elevated tenascin C levels suggests major ECM remodeling in these regions. As tenascin C has antiadhesive properties, we tested previously in a tenascin C knockout mouse model, whether it is the driver for fibronectin fiber relaxation in tumor tissues, however, the fibronectin fibers lost their tension in tumor tissues even in the absence of tenascin C^31^. Regarding direct cell-matrix interaction, we detected multiple integrins (e.g. Itga5, Itgb1, Itgav, Itgb3), none exhibited a significant change in abundance in the FnBPA5-positive region. Nonetheless, integrins may still play a role, potentially being regulated at the level of localization and activation rather than expression. Another consideration is that the increased tissue damage in these spatially defined areas, potentially due to granuloma-like structures, may cause the FnBPA5 signal, indicating irreversible tissue destruction.

Understanding the functional consequences of fibronectin fiber relaxation in neutrophil clusters is of particular interest, as stretched fibronectin fibers are indicative of healthy organs^30^. Release of this tension may have implications locally or systemically, potentially impacting immune responses and pathogen clearance. However, this remains technically challenging, and we have not been able to test any hypotheses relating to the functional consequences of fibronectin fiber tension changes in this work. Global deficiency in fibronectin is not viable and good systems to acutely locally deplete fibronectin do not currently exist. An alternative would be to examine the role of neutrophils through depletion experiments. However, this approach is not feasible due to the inherent limitations of the biological model. Neutrophil depletion is lethal in most mice by day 2 post infection due to the critical role in defense against *S*. Tm. Due to the severity of the model, it also is unsuitable to study disease resolution. Alternative mouse models with lower susceptibility to *S*. Tm infection could be considered but they would require recharacterization of ECM remodeling in detail due to differences in disease kinetics and severity in these mice^33^. Advances in organoid or organ on a chip technologies may provide ethical ways to probe functional consequences in the future. We are hopeful that technical advancements in mass spectrometry technology and optimization of TAILS protocols will eventually allow the use of LCM-collected material as input, which is currently not feasible. Since neutrophils are likely to recruit other immune cells, such as macrophages to their maturing clusters, studying the impact of macrophages on fibronectin fiber relaxation and ECM remodeling in general would be very interesting.

In summary, this study represents a significant step in unraveling the intricacies of cecal proteome and ECM dynamics during *S*. Tm infection on a tissue layer basis. The synergistic approach of combining LCM with low biomass LC-MS/MS proteomics enriched by a mechanobiological perspective charts a novel trajectory for future investigations and provides a foundation for translational applications in infectious disease management.

## Methods

### Ethics statement

All animal experiments were approved by the legal authorities (license ZH120/19) Kantonales Veterinäramt Zürich, Switzerland) and performed according to the legal and ethical requirements. Animals were scored daily for any expected or unexpected adverse events. Humane endpoints were defined in the license. Special focus was put on the 3 R principles (Replacement, Reduction and Refinement) for humane animal handling.

### Mice

Male and female specific-pathogen-free C57BL/6 J mice (10-11 weeks old) from an inbred colony at the ETH Phenomics center were used for all experiments. Animals were housed in groups of 2-5 animals in individually ventilated cages in the ETH Phenomics center (EPIC, RCHCI), ETH Zurich with ad libitum access to water and food at all times. Before the experiments mice were accustomed to their handling and trained to obtain a fruit-peanut mix (3:2:3 of 20% maltose solution, peanut-oil (sterilized), and fruit puree (fruit puree containing apples (56%), bananas (30% and raspberry (14%), pasteurized)) from the micropipette once per day on two consecutive days. One day before the *S*. Tm infection, the mice were pretreated with 25 mg of streptomycin in 75 µl sterile fruit-peanut solution via voluntary feeding. The Salmonella culture to infect the mice was prepared as overnight culture from wild-type *Salmonella enterica* serovar Typhimurium clone SB300, a derivative of strain SL1344^33^, grown for 12 h in Lysogeny Broth (LB) medium containing 50 µg/ml streptomycin at 37 °C and 180 rpm. Then the bacterial culture was diluted 1:20 in fresh LB medium without antibiotics and the subculture was grown for 3 h at 37 °C and 180 rpm. To prepare the fruit-peanut suspension, the bacteria were washed twice in cold PBS before adding. Mice were euthanized by CO2 asphyxiation followed by blood withdrawal from the heart at the indicated timepoints and tissue samples were collected for further processing. The mock-infected control group was euthanized on day 3, together with the 72 h p.i. group. No anesthesia of the mice was carried out in any of the experiments. To avoid cage effects, the individual groups were formed by mice from multiple cages.

### Analysis of *S*. Tm loads in intestines, mesenteric lymph nodes, spleens, and livers

Fresh fecal pellets, cecal content, spleen and liver samples were collected, weighed, and placed in 1 ml of PBS. They then were homogenized by bead beating (3 mm steel ball, 25 Hz, for 1.5 min using a Tissue Lyser (Qiagen, Germany)). Numbers of CFU were determined by plating appropriate dilutions on Mc-Conkey agar plates (with streptomycin at 50 µg/ml) and incubating them overnight at 37 °C.

### Immunohistochemical procedure

Co-stainings for relaxed (Cy5.5-FnBPA5) and total fibronectin fibers as well as with anti-Ly6B.2 antibody (#MCA771G, BioRad, Hercules, California, USA) were performed as follows: Tissue cryosections (20 µm thick) on glass slides were encircled with a hydrophobic pen (H-4000, Vector Laboratories, USA) to decrease staining solution usage. For all following individual steps, 100 µl of solution was used per cryosection. The tissues were then washed once with PBS and blocked with 4% BSA in PBS for 30 min. Cy5.5-FnBPA5 solution was diluted in PBS, added to the sections (5 µg/ml) and incubated for 1 h. The sections were then washed by immersing the whole glass slide into a beaker with PBS (3×5 min each). In the next step, the tissue sections were fixed with 4% formaldehyde solution (#P087.3, ROTI®Histofix, Carl Roth) for 10 min and subsequently washed with PBS 3x for 5 min each. Then sections were blocked for 45 min using a blocking buffer containing 5% goat or donkey serum (depending on the host species of the secondary antibody) (#G9023 and #D9663, Merck) and 0.3 M glycine (#56-40-6, Merck) in PBS. The collagen hybridizing peptide (R-CHP, 3Helix, Salt Lake City, UT, USA) staining was performed according to the company’s protocol^86^. Fibronectin was stained using a polyclonal anti-fibronectin antibody (ab23750, Abcam, Cambridge, UK), collagen I was stained using a polyclonal anti-collagen I antibody (ab34710, Abcam) and neutrophils were stained using an anti-Ly6B.2 antibody all at a dilution of 1:100 incubating over night at 4 °C in a humidified chamber. After 3 washes with PBS (5 min each), the secondary antibody goat anti-rabbit IgG Alexa 488 (A11043, Thermo Fisher Scientific) or goat anti-rat IgG Alexa 488 (ab150157, abcam) at 1:200 was applied for 1 h at room temperature. After a quick wash with PBS, co-stain with 4′,6-diamidino-2-phenylindole (DAPI) (D9564, Sigma Aldrich) was performed (10 µg/ml) for 10 min. Last, the sections were washed 3x for 5 min in PBS, dried and mounted using ProLong Gold antifade mounting medium (#P36930, Thermo Fisher Scientific). Mounted and stained sections were allowed to dry at room temperature and stored at 4° C before image acquisition.

### Microscopy

All immunohistochemistry stains were imaged using the Nikon Eclipse Ti2 microscope, equipped with the Yokogawa Confocal Scanner Unit CSU- W1-T2 and operated with the NIS-Elements Software. Images were acquired using either a 20×0.75 CFI Plan Apo λ or a 60×1.2 CFI Plan Apo VC Water objective. SHG images were acquired using a Leica SP8 multi-photon microscope with an excitation at 880 nm.

### Quantification of FnBPA5 signals

FnBPA5 signals were analyzed using a pixel-by-pixel approach. First, manual annotation in ImageJ and QuPath were made for muscosal and muscularis externa areas. Second, all pixels above the corresponding scrFnBPA5 signal were counted and their intensity was measured. For the analysis of FnBPA5-positive pixels, all xy coordinates plus intensity values were extracted from the images and separately treated for the mucosal and the muscularis externa areas. Then the percentage of positive pixels was calculated from all mucosal or muscularis externa pixels defined in the manual annotations. Positive pixel counts as well as the intensity distribution were visualized using python. To calculate Ly6B.2-specific signal, a QuPath algorithm was used to detect Ly6B.2 clusters and then the FnBPA5-positive pixel coverage was calculated in these clusters versus the rest of the mucosa.

### Mass spectrometry sample preparation and processing of LCM material

The MMI CellCut Laser Capture Microdissection device (Molecular Machines & Industries) was used to perform the Laser Capture Microdissection. 20 µm cryosections were captured on MMI membrane slides (MMI Prod. No. 50103) and subsequently stained with H&E using the MMI H&E Staining Kit Plus (MMI Prod. No. 70302). Collection of the microdissected tissue areas (in total 500.000 µm^2^) was done with the mmi isolation cap tubes (200 µl) and tissues were kept at -20° C until further processing. Tubes were turned upside down fixed in this position and the sample lysis was performed directly on the tube lid. Tubes were opened and 10 µl RIPA buffer (25 mM Tris•HCl pH 7.6, 150 mM NaCl, 1% NP-40, 1% sodium deoxycholate, 0.1% SDS (Cat# 89900 Thermo Scientific)) were added on top of the tissue on the lid itself. 1 µl of 10x TCEP (Tris(2-carboxyethyl)phosphine hydrochloride, #C4706, Merck) was added to the lid and mixed carefully by pipetting up and down. 1 µl of CAA (400 mM 2-Chloroacetamide, #C0267, Merck) was added, mixed carefully by pipetting and the tubes were closed to minimize evaporation. The tissues were incubated for 30 min at room temperature. Using a tabletop centrifuge the liquid was spun down and 48 µl ice-cold 100% acetone was added. This was followed by overnight incubation at −20 °C. After acetone precipitation, the lids containing isolation caps were exchanged manually with normal lids to avoid detachment of the silicone inlets during high-speed centrifugation. Samples were then centrifuged for 5 min at 21.000 x g. The acetone was removed, and the samples were air-dried in a fume hood. After that, the samples were resuspended in 10 µl 4 M GdCl (Guanidinium Hydrochloride) in 50 mM HEPES (#G3272, Merck) and sonicated in a water bath sonicator for 10 min. This was followed by a dilution in 30 µl 50 mM HEPES (pH 8.5) for digestion, LysC was added in a 1:50 ratio and samples were incubated for 4 h at 37 °C. Afterwards samples were diluted in another 30 µl 50 mM HEPES (pH 8.5), trypsin was added in a 1:10 protease:protein ratio (w/w) and samples were incubated overnight at 37 °C at 350 rpm. Using trifluoroacetic acid at a final concentration of 1% samples were acidified and the pH was verified using pH strips. After this the samples were centrifuged at 21.000 x g for 15 min and transferred onto EvoTip Pure trap columns for desalting and directly loading the samples onto the LC-MS. The EvoTip Pure tips were used according to the manufacturer’s instructions. In brief, Evotips were rinsed with 20 µl Solvent B (centrifugation at 800 g for 60 s). After this they were soaked in propanol until the Evotips turned pale white and equilibrated by soaking in 20 µl Solvent A (centrifugation at 800 g for 60 s). After loading the samples onto the wet Evotips, they were centrifuged again at 800 g for 60 s and washed with 20 µl Solvent A (centrifuged at 800 g for 60 s). Then, 100 µl Solvent A was added and the Evotips were centrifuged at 800 g for 10 s only.

### Data independent acquisition mass spectrometry analysis

Samples were placed on the EvoSep One liquid chromatography system (EvoSep, Denmark) and measured in-line with an Orbitrap Exploris 480 mass spectrometer (Thermo Fisher Scientific) coupled to a FAIMSpro device. Peptides were loaded on a EV1106 C18 column (15 cm × 150 µm, 1.9 µm diameter) and separated with the Whisper100 nanoflow and a 20SPD (samples per day) method consisting of a 58-minute gradient. Eluting peptides were injected to the mass spectrometer using a 20 µm fused silica emitter (EV1087), at a static voltage of 2300 V, carrier gas flow of 3.6 L/min and 240 °C ion transfer tube temperature and a positive polarity. A single compensation voltage of −45 V was applied to the FAIMS device during acquisition with a high-resolution MS1 (HRMS1) data-independent acquisition method. MS1 scans were recorded in the orbitrap detector at a 120.000 resolution, with a scan range of 400-1000 m/z, normalized AGC target of 300%, and injection time set to automatic. MS2 scans were recorded over the full m/z range with an isolation window of 8 m/z and 1 m/z window overlap. Peptides were fragmented using HCD (high collision dissociation), with a fixed normalized collision energy of 32%. The orbitrap resolution was set to 60.000 with first mass of 200 m/z. Normalized AGC target was set to 1000%, with maximum injection time set to automatic. MS1 scans were interspersed every 24 scan events (loop count 24), splitting the m/z range in three equal, 200 m/z parts. The raw data were searched with DIA-NN version 1.8, using a library-free (directDIA) approach and MS1 level quantification. The reference proteome was the mouse proteome database obtained from Uniprot (UP000000589 reviewed, accessed 17/06/2022). Precursor FDR was set to 1%, while Met N-terminal excision, Met oxidation and C carbamidomethylation were added as modifications. Match between runs and RT-dependent cross-run normalization were set to True. Trypsin/P was used as the protease, with one allowed missed cleavage, with otherwise default settings. Search results and protein quantification tables were used for further post-processing and analysis. To note, protein groups are denoted as proteins throughout the text, aiming to enhance the fluidity of reading.

### TMT-TAILS sample preparation

The cecum samples used were embedded in cryomedium for other experimental design reasons and first had to be cleaned thoroughly from the freezing medium. First, 500 µl of homogenization buffer (4 M GuHCl, 0.1 M EDTA, 1:10 protease inhibitor in MilliQ) was added to the samples and samples were placed in a Bioruptor® Pico sonication device (Diagenode, Hologic Inc, Belgium) for 45 cycles (30 sec on, 30 sec off). Then ice-cold TCA (trichloroacetic acid) was added to a final concentration of 20% and incubated for 20 min on ice. Samples were centrifuged at 16.000 g for 20 min at 4 °C and the supernatant was discarded. Then, 4 washes with ice-cold 10% TCA were performed (centrifugation for 5 min at 16.000 g to pellet the samples in-between). Subsequently, the samples were washed with 1 ml ice-cold 100% acetone for another 4 times in total. After the last wash, the samples were air-dried. After this they were resuspended in 20 µl of 0.2 M NaOH and incubated for 2 min at room temperature with 350 rpm. Then, 80 µl TAILS buffer (250 mM HEPES, 2.5 M GuHCl) was added to the samples and the pH was measured to match a pH of 7.8. Further sample processing was performed as published in a detailed methods paper^87^. In short, reduction of cysteine residues was performed by adding TCEP to a final concentration of 5 mM and incubation of samples at 65 °C for 45 min with 400 rpm. Then, cysteine residues were alkylated by addition of CAA to a final concentration of 20 mM and another incubation step at 65 °C at 400 rpm for 30 min. To prepare TMT reagent, 200 µg aliquots were dissolved in 110 µl of DMSO and mixed with equal amount of sample by pipetting. Samples were incubated for 1 h at room temperature. Then, to quench the labeling reaction ammonium bicarbonate was added to a final concentration of 100 mM and mixed by vortexing, followed by 30 min incubation at room temperature. Subsequently, all labeled samples with the individual TMT reagents were mixed in a 15 ml conical tube. Then, 8 times the sample volume of ice-cold acetone and 1 time the sample volume of ice-cold methanol were added, and the samples were incubated at −80 °C for at least 2 h. Then, the samples were centrifuged at 4500 x g at 4 °C for 20 min and the supernatant was discarded. For further washing 5 ml ice-cold methanol was added and again centrifuged at 4500 g at 4 °C for 20 min. After discarding the supernatant, the samples were air-dried. The samples were then resuspended in 100 mM NaOH at a ratio of 10:1 (w/v) protein to NaOH and mixed well by pipetting. Then, MilliQ water was added at protein:MilliQ ratio of 5:4 (w/v) and 1 M HEPES at a ratio of 10:1 (w/v). The samples were subsequently transferred into low binding tubes. For digestion, trypsin was added at protein:trypsin ratio of 50:1 (w/w) and the samples were incubated overnight (at least 16 h, max. 24 h) at 37 °C with 350 rpm. 10% of the digest was taken out as non-pullout sample and stored at −20 °C until desalting. For the remaining samples, the pH was adjusted to pH 6.5 using 2 M HCl and the HPG-ALD polymer was added in a protein:polymer ratio of 1:4 (w/w). Then, cyanoborohydride was added to a final concentration of 50 mM and samples were incubated at 37 °C for 16 h. On the next day 30 kDa Amicon Ultra-0.5 ml centrifugal filter units were prepared with 400 µl MilliQ water following the manufacturer’s instructions and centrifuged at room temperature. Then, the samples were transferred to the filter unit and centrifuged at 10.000x *g* for 10 min at RT. The filters were washed with 100 µl of 100 mM ammonium bicarbonate and centrifuged again at 10.000 g for 10 min at RT. Both flowthroughs were combined, acidified with TFA to a final concentration of 1% TFA and further processed together with the NPO sample, which was brought to a final volume of 200 µl using resuspension buffer (2% ACN, 1% TFA). To prepare the desalting cartridges, the Sep-Pak got activated with 800 µl of methanol. Then, washed with 800 µl of elution buffer (80% ACN, 0.1% formic acid (FA), 19.9% MilliQ grade water) and equilibrated with 800 µl of equilibration buffer (3% ACN, 1% TFA) twice. Then the samples were loaded, and the cartridges were washed twice with 800 µl of washing buffer (0.1% formic acid). After theaddition of 200 µl Buffer B (ACN) the flowthroughs were collected, and the eluates were dried in a speed vacuum concentrator. Subsequently, the dried samples were resuspended in resuspension buffer and measured for peptide concentration. 500 ng of peptides were loaded on EvoTips similarly to LCM samples for subsequent LC-MS/MS analysis.

### TMT-TAILS mass spectrometry analysis

We measured the pre-TAILS and TAILS samples, containing the total proteome and the enriched N-terminome respectively, with the EvoSep One platform (Evosep, Denmark) in line with an Orbitrap Exploris 480 mass spectrometer, which was coupled to a FAIMSpro interface. Peptides were separated using a PepSep column (Bruker Daltonics, cat. no. 1895812) during an active gradient of 118 min (Whisper100 10SPD method). Peptides were injected into the mass spectrometer with a PepSep emitter (Bruker Daltonics, cat. no. 1893519) with a positive ion spray voltage of 2300 V, and an ion transfer tube temperature of 240 °C. The carrier gas flow was set to 3.6 L/min, and FAIMS was operated in standard resolution in 3 different compensation voltages (CVs) of −40, −55, and –70. Data acquisition was performed as follows across all CVs. MS scans were acquired in the Orbitrap at 120,000 resolution and a scan range of 375–1500, automatic maximum injection time, RF Lens of 60%, and normalized AGC target of 300%. We included filters of charge state between 2–7, dynamic exclusion with a duration of 60 sec with 10 ppm mass tolerance, minimum intensity of 5000 and precursor fit at 70% before MS/MS acquisition. Top 10 data dependent MS/MS scans were acquired for each CV as centroids in the Orbitrap, which was operated at 60,000 resolution. We used an isolation window of 0.7 m/z, first mass of 110, HCD fragmentation at 34% NCE, with automatic maximum injection time and normalized AGC target at 75%.

The raw data were analyzed with Proteome Discoverer v2.4. Both raw files were included in the same search as fractions and searched against the mouse proteome, using the same database as the LCM analysis. The peptide library was constructed with semi-tryptic specificity and 2 maximum missed cleavages. Methionine oxidation (+15.995) and asparagine deamidation (+0.984) were added as dynamic modifications. TMTpro (+304.207) and acetylation (+42.011) were added as dynamic modifications on the peptide and protein terminus, respectively. Cysteine carbamidomethylation (+57.021) and lysine TMTpro labeling were added as static modifications. Peptide-spectrum matching was performed with Sequest HT, and FDR control with Percolator with 1% strict, and 5% relaxed FDR cutoffs. Peptides and proteins were quantified using the Reporter Ion Quantifier node, normalized to the total peptide amount of each channel. The peptide group and protein reports were exported for downstream analysis with Python 3.8 and CLIPPER 2.0.

## Supplementary information

Supplementary Information

Supplementary Data 1

Supplementary Data 2

Supplementary Data 3

Supplementary Data 4

## Data Availability

The mass spectrometry proteomics data have been deposited to the ProteomeXchange Consortium via the PRIDE^88^ partner repository with the dataset identifiers **PXD043256** and PXD056083 (LCM dataset of FnBPA5-positive regions in inflamed mucosa). Materials available on request to the corresponding author.
