# Supplementary material for: Relaxation of mucosal fibronectin fibers in late gut inflammation following neutrophil infiltration in mice
Source: NPJ Biol Phys Mech. 2025 Feb 4;2(1):4. doi: 10.1038/s44341-024-00006-y (PMC11794144; doi:10.1038/s44341-024-00006-y)
Supplement: Supplementary file 1 — Supplementary Information [file 44341_2024_6_MOESM1_ESM.pdf]

Relaxation of mucosal fibronectin fibers in late gut inflammation following neutrophil infiltration in mice

Ronja Rappold[1,3*], Konstantinos Kalogeropoulos[2*], Gianna La Regina[3], Ulrich auf dem Keller[2], Emma Slack[3,4,#], Viola Vogel[1,4,#]

[1] Institute of Translational Medicine, ETH Zurich, Zurich, Switzerland
[2] Department of Biotechnology and Biomedicine, Technical University of Denmark, Kgs. Lyngby, Denmark
[3] Institute of Food, Nutrition and Health, ETH Zurich, Zurich, Switzerland
[4] Botnar Research Center for Child Health, Basel, Switzerland

***** These authors contributed equally to this work and share first authorship.
[#] Emma Slack and Viola Vogel share senior authorship.

Supplementary Information

Supplementary Figure 1: Bacterial counts in the cecum, distal colon, mesenteric lymph nodes, spleen, and liver of C57BL/6 mice infected with *S.* Tm (magenta) and mock-infected control (green) mice.

Supplementary Figure 2: Relative abundances of proteins specific for mucosa and muscularis externa from laser capture microdissection coupled LC-MS/MS analysis.

Supplementary Figure 3: Comparable distribution of matrisome subcategories in the four groups healthy mucosa, inflamed mucosa, healthy muscularis externa, and inflamed muscularis externa.

Supplementary Figure 4: Mucosal FnBPA5 and SHG signal on day 3 p.i. with *S.* Tm in C57BL/6 mice shows no spatial correlation between collagen bundles and relaxed fibronectin fibers.

Supplementary Figure 5: Mucosal FnBPA5 and collagen signal on day 3 p.i. with *S.* Tm in C57BL/6 mice.

Supplementary Figure 6: Image analysis method and quantification of Ly6B.2-positive clusters as well as locally defined FnBPA5 intensities.

Supplementary Figure 7: Additional representative images of neutrophil clusters and the spatially correlated relaxed fibronectin fibers on day 3, but not yet on day 2.

Supplementary Figure 8: TAILS analysis of cecum tissue from *S.* Tm infected (day 3 p.i.) and mock-infected C57BL/6 mice.

Supplementary Data 1: List of proteins detected solely in the inflamed mucosa.

Supplementary Data 2: List of proteins detected solely in the inflamed muscularis externa.

Supplementary Data 3: List of proteins identified as significantly differentially expressed in the inflamed mucosa compared to the healthy mucosa .

Supplementary Data 4: List of proteins identified as significantly differentially expressed in the inflamed muscularis externa compared to the healthy muscularis externa.

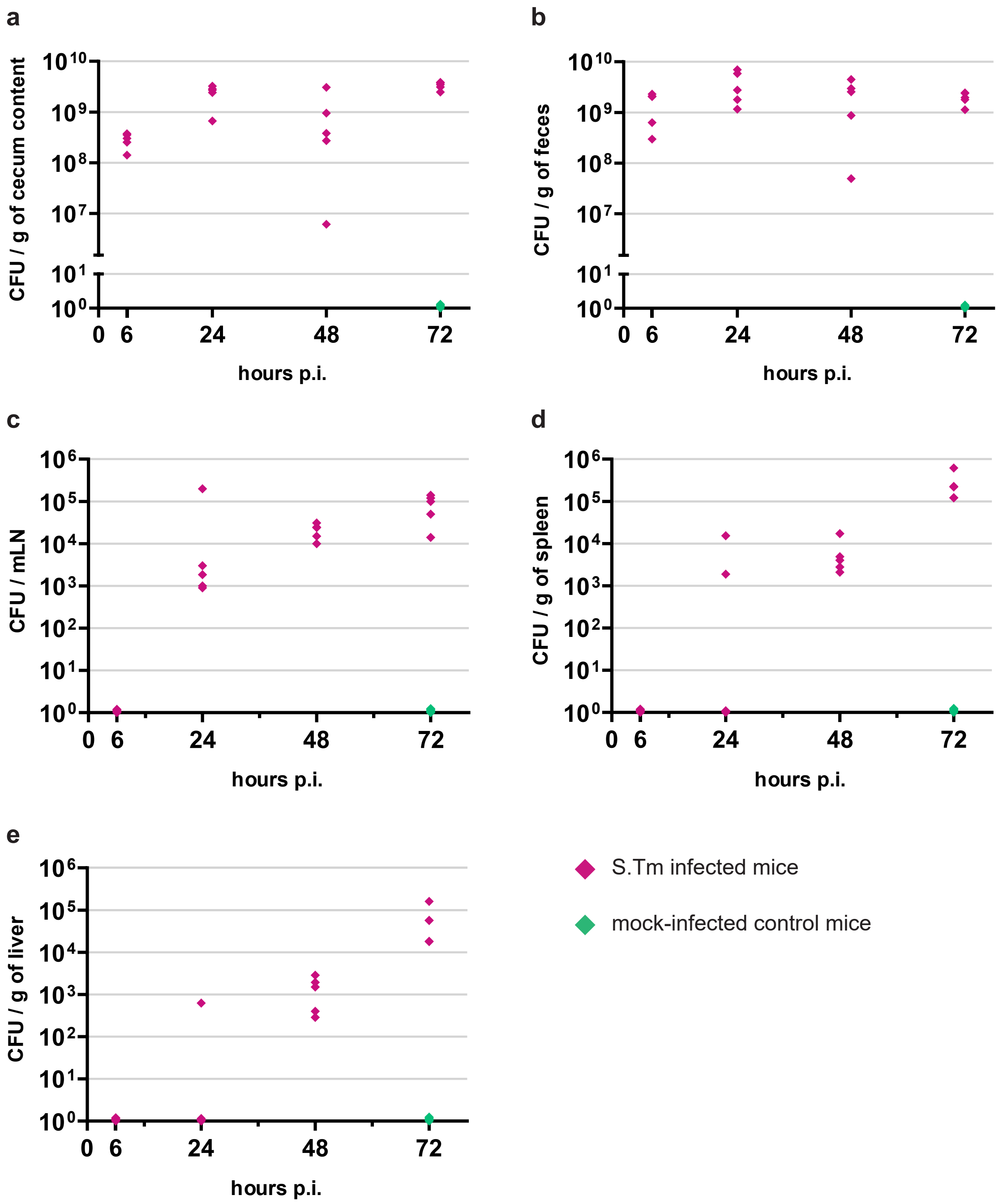

Supplementary Figure 1: Bacterial counts in the cecum, distal colon, mesenteric lymph nodes, spleen, and liver of C57BL/6 mice infected with *S.* Tm (magenta) and mock-infected control (green) mice. Colony forming units (CFU) were determined in the cecum content (a), feces (**b**), mesenteric lymph nodes (mLN) (**c**), the spleen (**d**), and the liver (**e**) at the indicated timepoints post infection (p.i.). Marker points represent individual mice (n=5 in each group).

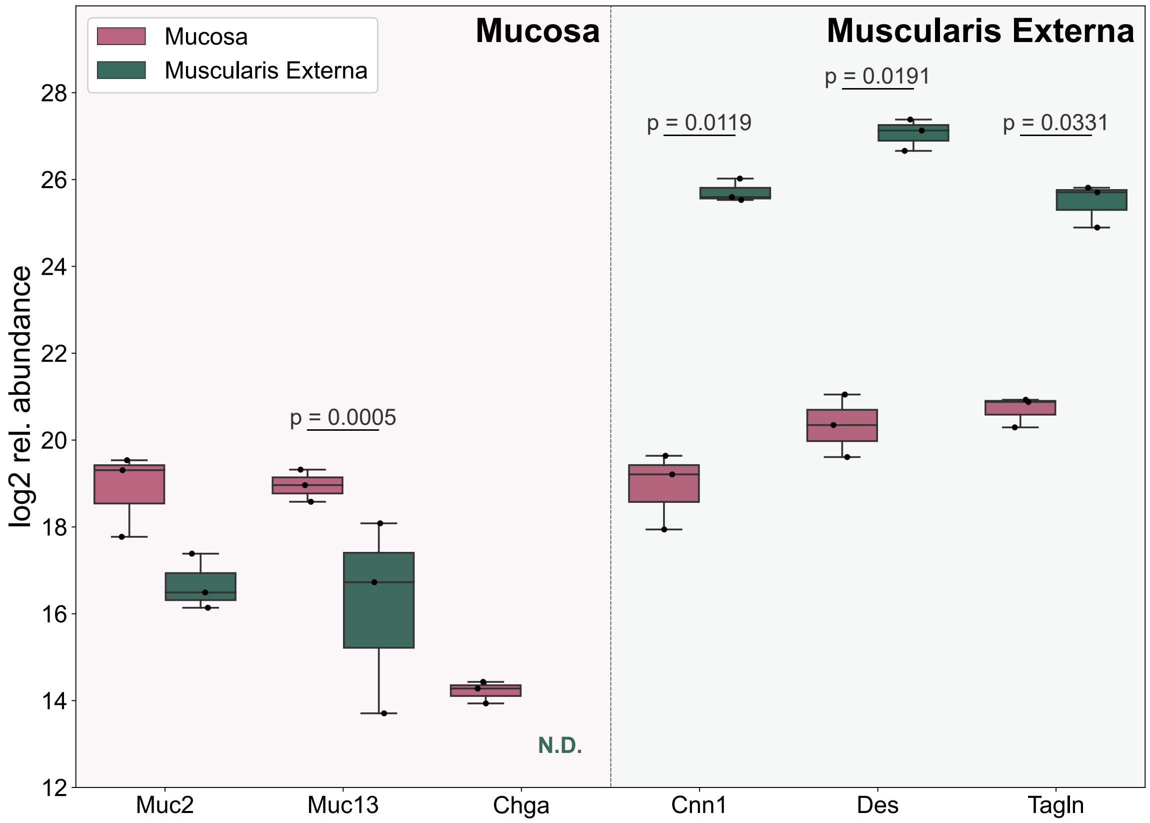

Supplementary Figure 2: Relative abundances of proteins specific for mucosa and muscularis externa from laser capture microdissection coupled LC-MS/MS analysis. Mucosal proteins: mucin2 (Muc2), mucin13 (Muc3) and chromogranin A (Chga). Muscularis external specific proteins: calponin1 (Cnn1), desmin (Des) and transgelin (Tagln). Boxplots extend from first quartile to third quartile with median abundance indicated as horizontal line and marker points representing individual mice (n=3). Statistical analysis: two-sided paired Student's t-test. P-values indicated.

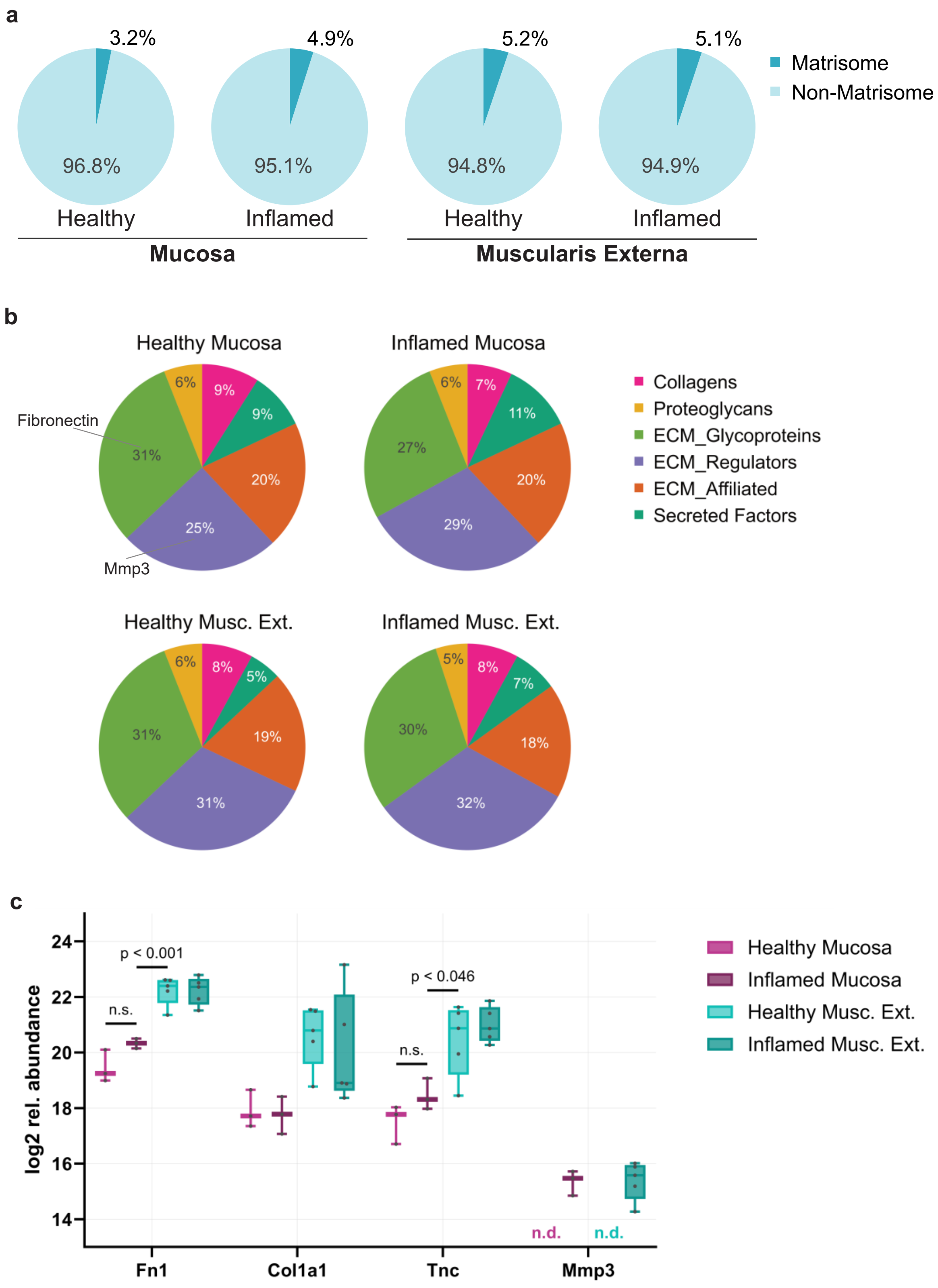

Supplementary Figure 3: Comparable distribution of matrisome subcategories in the four groups healthy mucosa, inflamed mucosa, healthy muscularis externa, and inflamed muscularis externa. **a:** Matrisome related protein percentages of all detected proteins in the four different conditions were between 3-5%. **b:** The distributions of matrisome subcategories in the four different conditions are similar between health states and tissue layers. **c:** Log2 relative abundance of the proteins of interest fibronectin (Fn1), collagen I (Col1a1), tenascin C (Tnc) and matrix metalloproteinase 3 (Mmp3) reflect differences in

abundance in tissue layers and health state. Boxplots extend from first quartile to third quartile with median indicated as horizontal line and marker points representing individual mice (n=3-5). Statistical analysis: One-way ANOVA with Tukey's multiple comparison test, p-values indicated. Musc . Ext, muscularis externa.

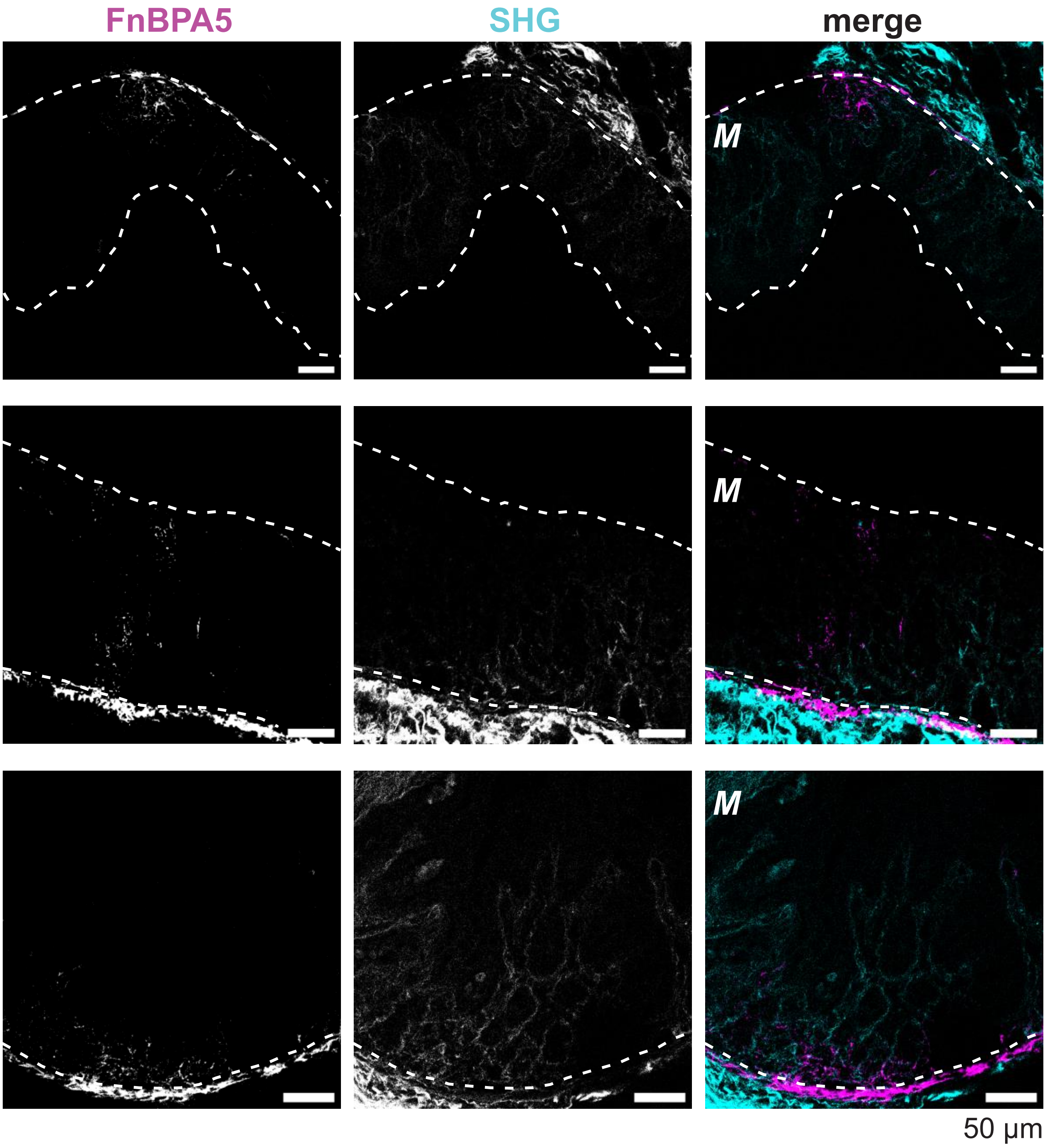

Supplementary Figure 4: Mucosal FnBPA5 and SHG signal on day 3 p.i. with *S.* Tm in C57BL/6 mice shows no spatial correlation between collagen bundles and relaxed fibronectin fibers. Each row represents a region of interest with a cluster of relaxed fibronectin signal (magenta) and the corresponding SHG signal (cyan). White dashed lines indicate mucosal areas in the cecal cross-sections. Scale bar: 50 µm. *M*: mucosa.

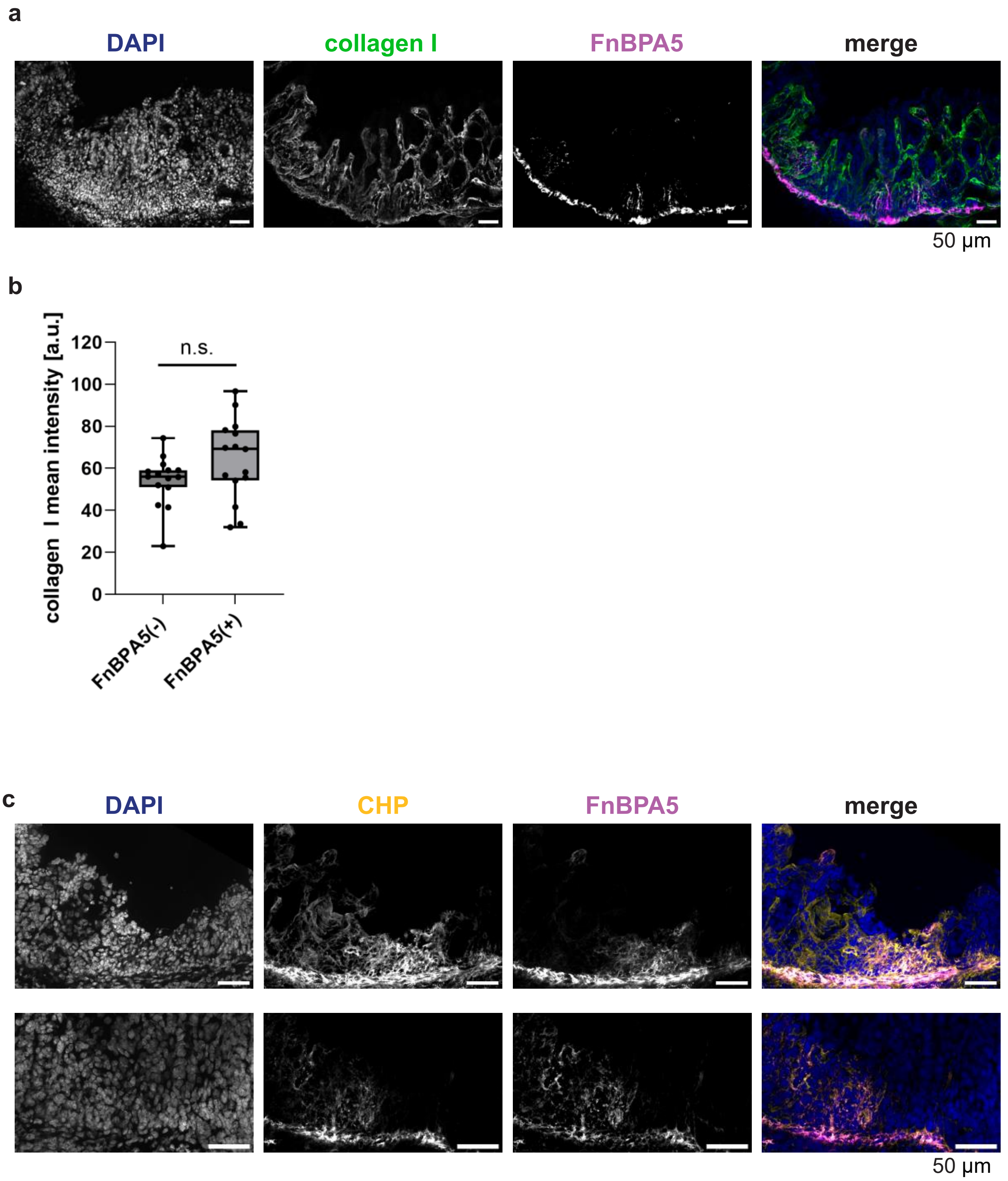

Supplementary Figure 5: Mucosal FnBPA5 and collagen signal on day 3 p.i. with *S.* Tm in C57BL/6 mice. a: Immunohistochemistry co-stainings of collagen I and FnBPA5 show no spatial correlation between collagen I and relaxed fibronectin fibers in the inflamed mucosa. Cryosections (day 3) were stained against cell nuclei using DAPI (blue), collagen I using a polyclonal anti-collagen I antibody (green) and relaxed fibronectin fibers using the FnBPA5 peptide (magenta). **b:** Quantification of collagen intensity in FnBPA5-positive regions vs other regions in the inflamed mucosa yields no significant increase in collagen I signal related to areas of relaxed fibronectin fibers. Boxplots extend from first quartile to third quartile with median indicated as horizontal line and marker points representing individual regions of interest analyzed (n=15). Statistical analysis: One-way ANOVA with Tukey's multiple comparison test, n.s.: $p \geq 0.05$. **c:** Immunohistochemistry co-stainings of collagen hybridizing peptide representing denatured collagen and FnBPA5 show an increase in collagen remodeling in areas with relaxed fibronectin fibers in the inflamed mucosa.

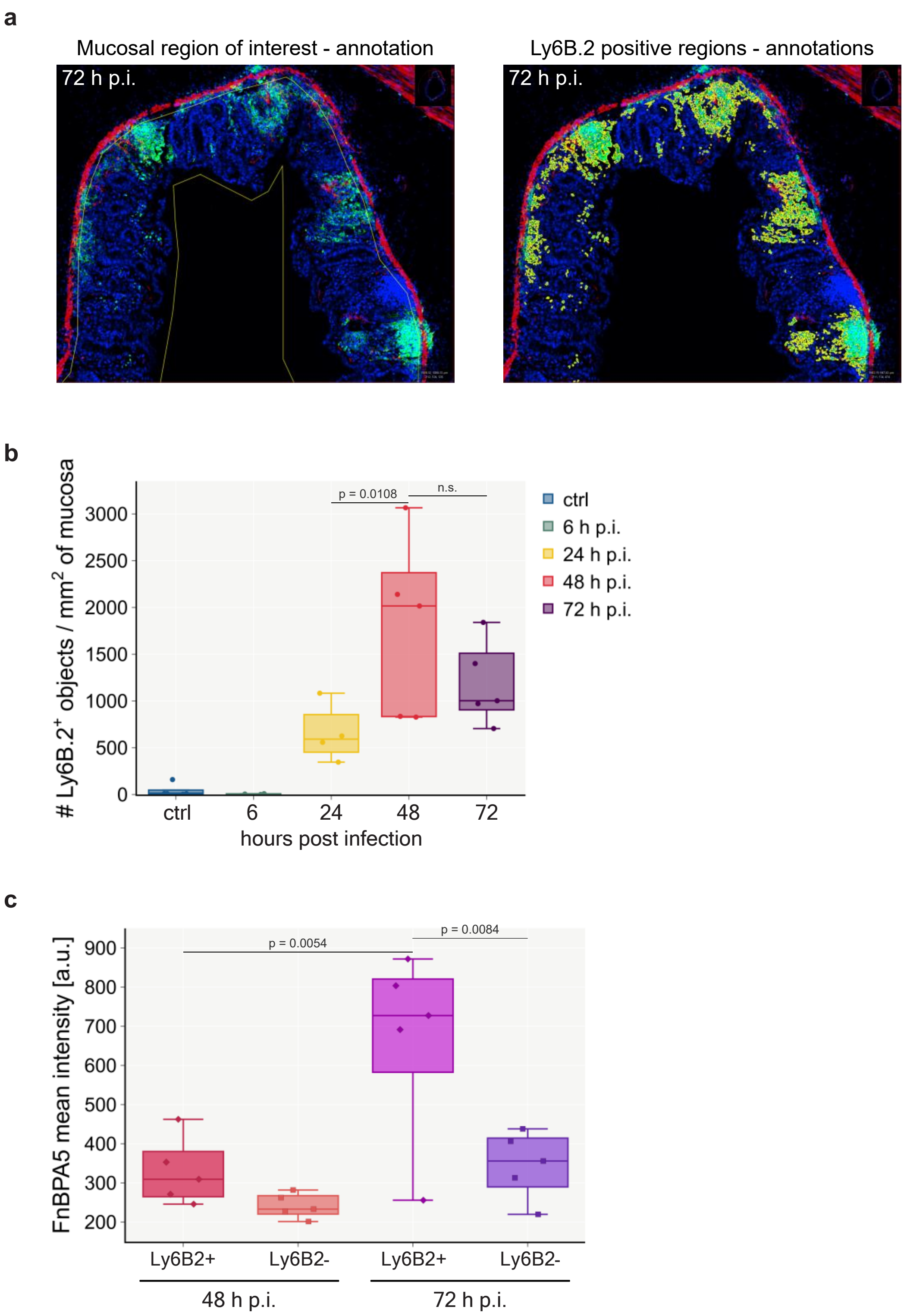

Supplementary Figure 6: Image analysis method and quantification of Ly6B.2-positive clusters as well as locally defined FnBPA5 intensities. a: Manual annotation of the mucosal region (left) and automated detection of Ly6B.2-positive objects (right) indicated by yellow outlines. **b:** Quantification of Ly6B.2-positive objects per area in the mucosa during the timecourse of the *S.* Tm infection. **c:** Quantification of FnBPA5 mean intensity in Ly6B.2-positive vs. Ly6B.2-negative regions on day 2 and day 3 post infection (p.i.) shows significantly higher intensity between the two regions of interest on day 3 but not yet on day 2. b-c: Boxplots extend from first quartile to third quartile with median indicated as horizontal line and marker points representing individual mice (n=3-5). Statistical analysis: One-way ANOVA with Tukey's multiple comparison test, p-values indicated, n.s.: $p \geq 0.05$.

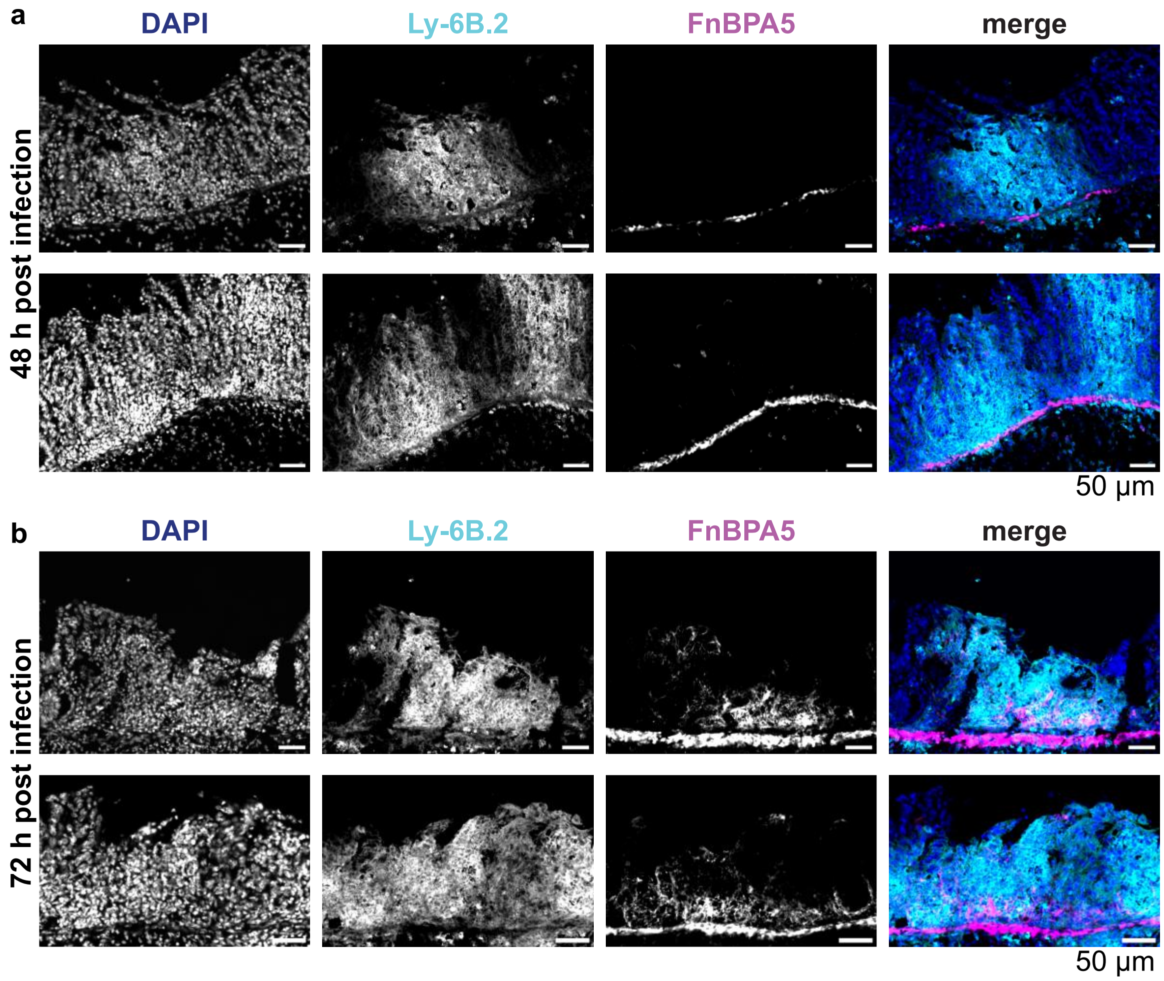

Supplementary Figure 7: Additional representative images of neutrophil clusters and the spatially correlated relaxed fibronectin fibers on day 3, but not yet on day 2. Additional zoomed-in images of cecal cryosections stained for neutrophils using an anti-Ly6B.2 antibody (cyan), Cy5.5-FnBPA5 (magenta) as well as cell nuclei using DAPI (blue) showing no relaxed fibronectin fibers in the areas of neutrophil clusters on day 2 post infection (**a**), whereas strong spatial correlation is observed on day 3 post infection (**b**).

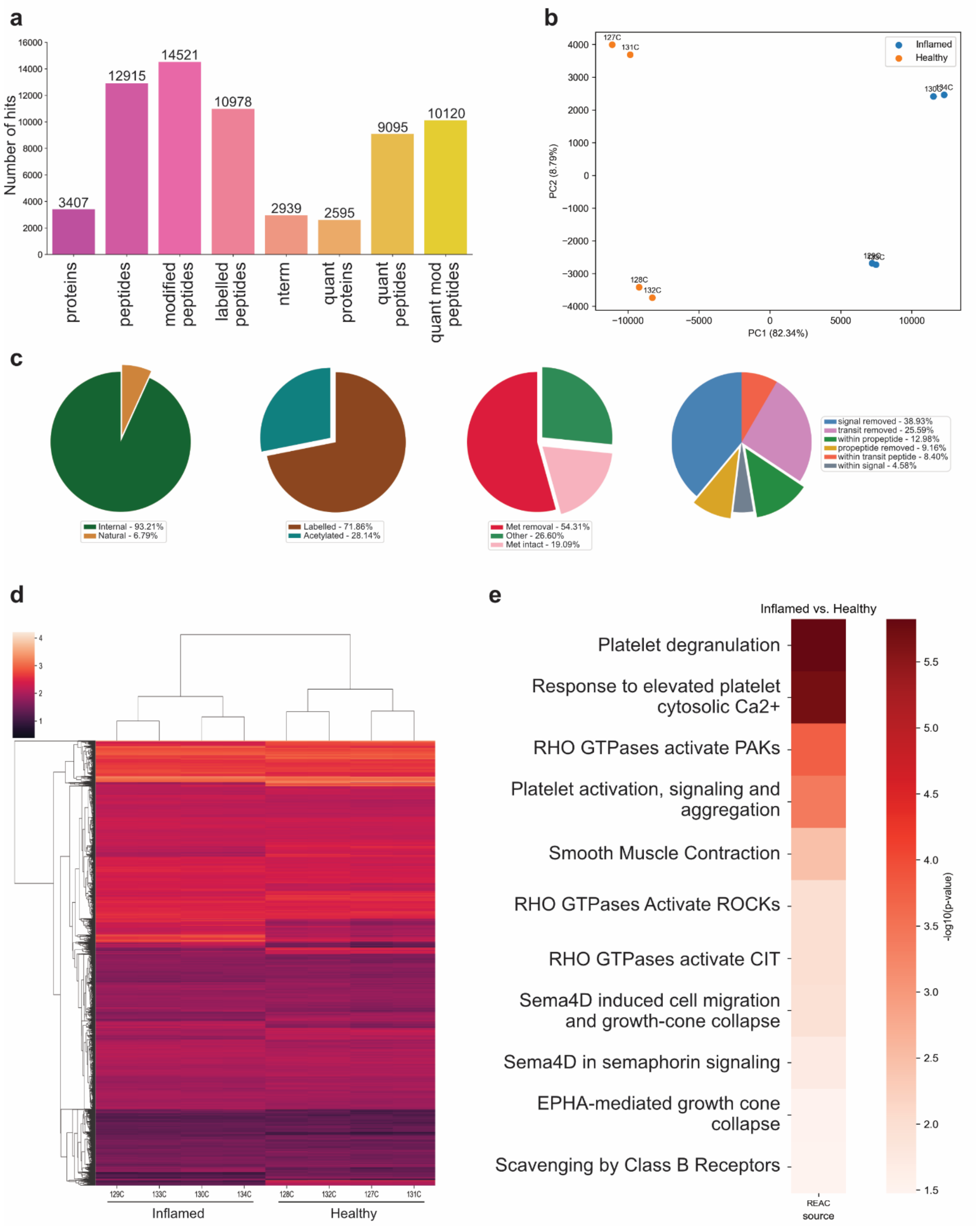

Supplementary Figure 8: TAILS analysis of cecum tissue from *S.* Tm infected (day 3 p.i.) and mock-infected C57BL/6 mice. a: Numbers of proteins, peptides and N-termini identified with high confidence are shown. **b:** PCA analysis separates the healthy and inflamed tissues. **c:** Distribution of N-termini and their post-translational modifications of peptides and proteins identified after N-terminal enrichment including distribution of acetylated versus non-acetylated N termini. **d:** Cluster map visualizing differences in the N-terminome between the healthy and *S.* Tm infected cecum tissue. **e:** Reactome pathway enrichment analysis of upregulated N-termini in the inflamed condition. Met, methionine.
